# SIRT1 ubiquitination is regulated by opposing activities of APC/C-Cdh1 and AROS during stress-induced premature senescence

**DOI:** 10.1038/s12276-023-01012-1

**Published:** 2023-06-01

**Authors:** Sang Hyup Lee, Ji-Hye Yang, Ui-Hyun Park, Hanbyeul Choi, Yoo Sung Kim, Bo-Eun Yoon, Hye-Jeong Han, Hyun-Taek Kim, Soo-Jong Um, Eun-Joo Kim

**Affiliations:** 1 Department of Molecular Biology, Dankook University, Cheonan, 31116 Korea; 2 Department of Integrative Bioscience and Biotechnology/Institute of Bioscience, Sejong University, Seoul, 143-747 Korea; 3 Soonchunhyang Institute of Medi-bio Science (SIMS), Soonchunhyang University, 31151 Cheonan-si, Republic of Korea; 4 Department of Integrated Biomedical Science, Soonchunhyang University, 31151 Cheonan-si, Republic of Korea

**Keywords:** Senescence, Ubiquitylation

## Abstract

SIRT1, a member of the mammalian sirtuin family, is a nicotinamide adenosine dinucleotide (NAD)-dependent deacetylase with key roles in aging-related diseases and cellular senescence. However, the mechanism by which SIRT1 protein homeostasis is controlled under senescent conditions remains elusive. Here, we revealed that SIRT1 protein is significantly downregulated due to ubiquitin-mediated proteasomal degradation during stress-induced premature senescence (SIPS) and that SIRT1 physically associates with anaphase-promoting complex/cyclosome (APC/C), a multisubunit E3 ubiquitin ligase. Ubiquitin-dependent SIRT1 degradation is stimulated by the APC/C coactivator Cdh1 and not by the coactivator Cdc20. We found that Cdh1 depletion impaired the SIPS-promoted downregulation of SIRT1 expression and reduced cellular senescence, likely through SIRT1-driven p53 inactivation. In contrast, AROS, a SIRT1 activator, reversed the SIRT1 degradation induced by diverse stressors and antagonized Cdh1 function through competitive interactions with SIRT1. Furthermore, our data indicate opposite roles for Cdh1 and AROS in the epigenetic regulation of the senescence-associated secretory phenotype genes *IL-6* and *IL-8*. Finally, we demonstrated that pinosylvin restores downregulated AROS (and SIRT1) expression levels in bleomycin-induced mouse pulmonary senescent tissue while repressing bleomycin-promoted Cdh1 expression. Overall, our study provides the first evidence of the reciprocal regulation of SIRT1 stability by APC/C-Cdh1 and AROS during stress-induced premature senescence, and our findings suggest pinosylvin as a potential senolytic agent for pulmonary fibrosis.

## Aging: protecting a protein that promotes proliferation

Two opposing molecular mechanisms determine the stability of SIRT1, a protein that prevents cells from prematurely entering the ‘retirement’ state known as senescence. This state, in which cell division is halted and tissue repair and regeneration impeded, is a natural endpoint of aging but also arises from cellular damage and other stressors. South Korean researchers led by Eun-Joo Kim at Dankook University, Cheonan, and Soo-Jong Um at Sejong University, Seoul, have identified two proteins that govern the onset of senescence by affecting SIRT1’s stability. One promotes the addition of chemical modifications to SIRT1 that mark it for destruction, while the other prevents such modifications. The researchers further showed that a drug that tips the balance in favor of SIRT1 stabilization could prevent early senescence in an animal model of pulmonary fibrosis, a currently incurable lung disorder.

## Introduction

Cellular senescence is a state of permanent cell cycle exit that occurs when cells lose their replicative ability in response to various stress signals, such as telomere dysfunction, DNA damage, oxidative stress, and aberrant oncogenic activation^1,2^. Senescent cells are enlarged, exhibit a flattened morphology and increased senescence-associated β-galactosidase (SA-β-gal) activity, and secrete soluble factors related to the local tissue environment and inflammation; these characteristics comprise the senescence-associated secretory phenotype (SASP)^3–5^. Cells with the SASP exhibit irreversible growth arrest and apoptosis resistance and these processes are activated through the p53/p21 or p16/pRb signaling pathway, a major route of senescence^6,7^. Senescence is involved in the pathogenesis of age-related disorders, tumor suppression, and organismal aging^8,9^. Based on recent findings that the selective elimination of senescent cells increases the lifespan of mice, research has been undertaken concerning potential senolytic drugs^3,10,11^.

The anaphase-promoting complex or cyclosome (APC/C) is an evolutionarily conserved multisubunit E3 ubiquitin ligase with key roles in the G1 phase and mitosis that involve the degradation of major cell cycle regulators^12,13^. A subcomplex containing APC2 and APC11 serves as a cullin-RING catalytic core of APC/C^14,15^. The catalytic activity of APC/C is regulated by two coactivators, Cdh1 and Cdc20, which provide substrate specificity through various conformational changes upon binding during the cell cycle^16^. The expression level of Cdh1 is significantly downregulated in breast and colon cancer, and Cdh1 suppression causes genomic instability and susceptibility to spontaneous tumors^17–19^. In addition to its role in cell cycle control, Cdh1 induces premature cellular senescence in response to DNA damage, as revealed by recent studies, but the process by which Cdh1 contributes to stress-induced premature senescence (SIPS) remains unclear^20–22^.

SIRT1 is an NAD^+^-dependent class III deacetylase and the mammalian ortholog of yeast Sir2^23,24^. SIRT1 has crucial roles in diverse cellular processes, including aging, senescence, inflammation, apoptosis, and metabolism, by removing acetyl groups from histone and nonhistone proteins^25,26^. Recent reports have indicated that SIRT1 protein expression is downregulated in response to DNA damage^27^ and oxidative stress^28^ through E3 ligase (both MDM2 and CHFR)-mediated polyubiquitination and subsequent proteasomal degradation. Another study has indicated that SIRT1 protein expression decreases via lysosome-mediated autophagy-associated degradation during aging and diverse SIPS processes^29^. Conversely, SIRT1 stabilization is promoted by the ubiquitin hydrolases USP22 and USP7, which cleave polyubiquitin chains of SIRT1^30,31^. However, the molecular mechanism underlying the ubiquitin-dependent degradation of SIRT1 in cellular senescence and the role of E3 ligase activation in SIRT1 ubiquitination under stress conditions remain undetermined. Until recently, a protein that counteracts the E3 ubiquitin ligase-mediated attack of SIRT1 and thus prevents SIRT1 degradation in cellular senescence has not been identified. We previously found that AROS interacts with SIRT1 and promotes SIRT1 deacetylase activity to drive p53 inactivation^32^. However, the mechanism by which AROS regulates SIRT1 activity and the role of AROS in cellular senescence requires further investigation.

In the present study, we observed a significant reduction in the SIRT1 protein levels under various stress conditions and identified the APC/C coactivator Cdh1 as a novel E3 ligase of SIRT1 that promotes the ubiquitination of SIRT1 for proteasomal degradation. We revealed that AROS antagonizes Cdh1-mediated SIRT1 ubiquitination via competition with Cdh1 for SIRT1 binding and thereby suppresses SIPS. Further analysis indicated opposing roles for Cdh1 and AROS in the epigenetic regulation of SASP-related genes and the activity of pinosylvin during bleomycin-induced pulmonary senescence, which suggests that a novel mechanism finely controls SIRT1 stability during stress-induced senescence and provides a potential target for senolytic drugs in the treatment of lung fibrosis.

## Materials and methods

### Cell lines and culture

HEK293 and MCF-7 cells were grown in Dulbecco’s modified Eagle’s medium, A549 and HCT116 cells were grown in RPMI-1640 medium, and TIG-3 cells (Japanese Collection of Research Bioresources Cell Bank, Japan) were incubated in Minimum Essential Medium (all media from Welgene, Inc.) containing 10% fetal bovine serum and 1% antibiotic-antimycotic mix (Thermo Fisher Scientific). All the cells were maintained and cultured under 5% CO_2_ in a humidified chamber at 37 °C. To obtain stable cell lines, A549 cells were transfected with Flag, Flag-AROS, sh-Luc, or sh-AROS using Lipofectamine Plus reagent (Thermo Fisher Scientific). After 48 h, the cells were treated with 0.8 mg/ml G418 (Thermo Fisher Scientific) or 0.1 mg/ml hygromycin B (Thermo Fisher Scientific). Resistant colonies were selected for 2 weeks, and stable gene expression was detected via western blotting (WB) using anti-Flag or anti-AROS antibodies.

### DNA constructs and antibodies

The SIRT1 and AROS constructs used in this study were previously described^32^. Full-length Cdh1, Cdc20, APC2, and APC11 were amplified via polymerase chain reaction (PCR) and subcloned into the plasmids Flag, Myc-tagged pcDNA3 (Thermo Fisher Scientific), pEGFP-C3 (BD Biosciences) and pGEX4T-1 (GE Healthcare). The antibodies used included the following: SIRT1 (Santa Cruz Biotechnology, sc15404), AROS (Santa Cruz Biotechnology, sc-86209; Abcam, ab201091), Cdh1 (Abcam, ab3242), Cdc20 (Santa Cruz Biotechnology, sc13162), Cyclin B1 (Santa Cruz Biotechnology, sc245), APC2 (Thermo Fisher Scientific, RB-067), APC11 (Abcam, ab154546), p53 (Santa Cruz Biotechnology, sc-126), p21 (Santa Cruz Biotechnology, sc-6246), p16 (Calbiochem, NA29), green fluorescent protein (GFP; Santa Cruz Biotechnology, sc-8334), polyubiquitinated conjugates (poly-Ub; Enzo Life Science, BML-PW8805-0500), acetylated-lysine (Cell Signaling Technology, 9814), hemagglutinin (HA; Merck Millipore, 05-904), Myc (Merck Millipore, 05-724), Flag M2 (Sigma‒Aldrich, F1804), LSD1 (Abcam, ab17721), H3K9me2 (Abcam, ab1120), H3K9ac (Abcam, ab12179), α-SMA-Cy3 (Sigma, C6198), collagen type I (Abcam, ab34710), elastin (Abcam, ab21600), fibronectin (Abcam, ab2413), and β-actin (Santa Cruz Biotechnology, sc47778).

### Virus production

293GPG cells were transfected with the pBABE puro *H-RasV12* (Addgene, Plasmid #9051) viral vector. Viral suspensions were collected at five time points (at 2-day intervals). After the final collection, the viral suspension was filtered through a 0.45-μm filter and concentrated using the Retro-X concentrator system (Thermo Fisher Scientific). The concentrated virus was suspended in TNE buffer (50 mM Tris, 130 mM NaCl, and 1 mM ethylenediaminetetraacetic acid).

### Senescence-associated β-galactosidase (SA-β-gal) staining

First, 1 × 10^4^ cells were seeded in 6-well plates and treated with doxorubicin (Sigma‒Aldrich) for 1 h. The concentrations of doxorubicin were 0.1 μM for MCF-7 cells and 0.5 μM for A549 and TIG-3 cells. Four days after doxorubicin withdrawal, SA-β-gal staining analysis was performed as previously described^33^. SA-β-gal-positive cells were counted using a microscope. Slides of 8-μm mouse tissue sections were fixed with a fixative solution (1% formaldehyde, 0.2% glutaraldehyde, 2 mM MgCl_2_, 5 mM ethylenediaminetetraacetic acid, and 0.02% NP40) and stained with a staining solution containing 0.2 M citric acid/Na phosphate buffer (pH 6.0) and 20 mg/ml X-gal for 24 h at 37 °C. The level of SA-β-gal staining was analyzed using ImageJ software (NIH).

### Immunoprecipitation (IP) and WB

IP and WB assays were conducted as previously described^32^. For IP, lysates from transfected cells were incubated with the indicated antibodies overnight at 4 °C. After a further incubation of 4 h with A/G or A-agarose beads (Santa Cruz Biotechnology), the bound proteins were discharged from the beads by boiling and subjected to WB using the appropriate antibodies. For WB, lysates from the beads were separated through sodium dodecyl sulfate gel electrophoresis, transferred to a polyvinylidene fluoride membrane (Merck Millipore), and probed with the indicated primary antibodies. The blots were reacted with horseradish peroxidase-conjugated anti-mouse (Santa Cruz Biotechnology) or anti-rabbit (Jackson) secondary antibodies. The protein bands were detected using enhanced chemiluminescent reagents (iNtRON Biotechnology) and the ChemiDoc imaging system (Bio-Rad).

### Glutathione S-transferase (GST) pull-down assays

GST pull-down assays were conducted as previously described^32^. Briefly, GST-fused AROS and Cdh1 proteins were expressed in *Escherichia coli* and refined with glutathione-Sepharose beads (GE Healthcare). Flag-SIRT1 protein was translated in vitro using the TNT rabbit reticulocyte system (Promega). GST protein (2 μg) was incubated with 10 μl of SIRT1 protein. The bound proteins were visualized by WB using an anti-Flag M2 antibody (Sigma‒Aldrich, F1804).

### Cycloheximide (CHX) chase assays

HEK293 cells were transiently transfected with the indicated constructs. Twelve hours after transfection, the cells were incubated with 50 µg/ml CHX (Sigma‒Aldrich) for the indicated times to prevent de novo protein synthesis. The cells were then harvested and lysed for WB analysis.

### In vivo and in vitro ubiquitination assays

For the detection of SIRT1 ubiquitination in vivo, cells were treated with 10 μM MG132 (Calbiochem) for 12 h before harvest. Cell lysates were subjected to IP using an anti-SIRT1 antibody. The polyubiquitination of SIRT1 was detected via WB using an anti-poly-Ub antibody or anti-HA antibody. For the in vitro ubiquitination assay, a ubiquitination kit (Enzo Life Science) was used in accordance with the manufacturer’s protocol. Briefly, APC/C complexes were purified from HEK293 cells via IP using an anti-APC2 antibody. Purified APC/C complexes were mixed with in vitro-translated Flag-SIRT1 and Myc-Cdh1 and then incubated in 50 μl of reaction buffer containing 2.5 μM biotinylated ubiquitin, 100 nM E1, 2.5 μM UbcH5, 5 mM Mg-ATP, 5 mM dithiothreitol, and 20 U/ml inorganic pyrophosphate (Sigma‒Aldrich) at 37 °C for 1 h. The reaction was terminated via the addition of 50 μl of 2× nonreducing gel loading buffer. Ubiquitinated SIRT1 was detected by WB using anti-SIRT1 or anti-Flag antibodies.

### Immunohistochemical (IHC) staining and immunofluorescence (IF)

Mouse lungs were fixed with 4% paraformaldehyde and embedded in OCT compound (Sakura) to obtain frozen tissue specimens. Frozen sections (8 μm) were treated with 1% hydrogen peroxide (H_2_O_2_) to quench endogenous peroxidase activity and then incubated with 1% bovine serum albumin for 1 h. Subsequently, the slides were incubated with the indicated primary antibodies for 16 h at 4 °C and then treated with biotinylated anti-rabbit secondary antibody (Vector Laboratories) for 1 h at room temperature. The sections were reacted with VECTASTAIN ABC reagent (Vector Laboratories) for 1 h, and peroxidase substrate 3,3-diaminobenzidine solution (Sigma‒Aldrich) was added until optimal color developed. The sections were then counterstained with Mayer’s hematoxylin solution (Sigma‒Aldrich). Each staining image was quantified using ImageJ software (NIH). For histopathological analysis, hematoxylin and eosin (H&E) staining was performed according to standard protocols. Alveolar spaces were measured using ImageJ. For connective tissue staining, frozen tissue sections were stained using a trichrome staining kit (ab150686, Abcam) according to the manufacturer’s instructions. Immunofluorescence was performed according to a standard protocol. The following antibodies and dilutions were used for immunostaining: SMA-Cy3 (Sigma, 1:1000), collagen type I (1:500), elastin (Abcam, 1:1000), and fibronectin (Abcam, 1:500).

### Reverse transcription-quantitative PCR (RT‒qPCR)

Total RNA from MCF-7, A549, or TIG-3 cells was isolated using TRIzol reagent (Thermo Fisher Scientific). RNA was reverse transcribed using M-MLV RTase (Promega) and random primers (Promega). The cDNA products were amplified and analyzed using SYBR Green Master Mix and the LightCycler system (Roche). For cDNA amplification, the following primer pairs were used: *Cdh1*, 5′-TACTCCCTGTCTCCCGTCAG-3′ (forward) and 5′-GGTACAGGCACTCCACAGGT-3′ (reverse); *SIRT1*, 5′-TCGCAACTATACCCAGAACATAGACA-3′ (forward) and 5′-CTGTTGCAAAGGAACCATGACA-3′ (reverse); *AROS*, 5′-GGAAGACGAAGGCAATTCAGGC-3′ (forward) and 5′-TCGGTGAACACGGTGCC-3′ (reverse); *IL-6*, 5′-CCAGGAGCCCAGCTATGAAC-3′ (forward) and 5′-CCCAGGGAGAAGGCAACTG-3′ (reverse); and *IL-8*, 5′-TTGGCAGCCTTCCTGATTTC-3′ (forward) and 5′-TCTTTAGCACTCCTTGGCAAAAC-3′ (reverse). The expression levels of each gene were normalized to GAPDH as an internal standard.

### Chromatin immunoprecipitation (ChIP)

ChIP assays were performed as previously described^34^. Briefly, doxorubicin was added to concentrations of 0.2 μM for MCF-7 cells and 0.5 μM for A549 and TIG-3 cells. After 1 h, doxorubicin was removed from the culture medium. Four days later, ChIP assays were performed using antibodies against SIRT1, LSD1, H3K9me2, and H3K9ac. For quantitative PCR analysis, the following primer pairs were used: *IL-6* promoter region, 5′-AATGTGGGATTTTCCCATGA-3′ (forward) and 5′-GCTCCTGGAGGGGAGATAGA-3′ (reverse); and *IL-8* promoter region, 5′-GGTTTGCCCTGAGGGGATG-3′ (forward) and 5′-ACAGAGCTGCAGAAATCAGGAAGGCT-3′ (reverse).

### RNA interference

The target sequences of short hairpin RNA (shRNA) for Cdh1 and AROS were as follows: Cdh1#1, 5′- CCACAGGATTAACGAGAAT-3′; Cdh1 #2, 5′-GGAGCCAACTGGAGCGTGA-3′; AROS#1, 5′-GGCAATTCAGGCCCAGAAA-3′; and AROS#2, 5′-ACCTGAAGTTTCTGACCAG-3′. shRNA transfection was conducted using Lipofectamine Plus reagent (Thermo Fisher Scientific) or polyethylenimine (Polysciences). The knockdown of Cdh1, Cdc20, and AROS was verified by WB analysis.

### Animal studies

Six-week-old male C57BL/6J mice were obtained from DBL (Eumseong, Korea) and acclimatized to local vivarium conditions for 7 days. To induce pulmonary fibrosis, mice were injected with a single intratracheal dose of 1.25 units/kg bleomycin (Selleckchem). The control mice received an equal volume of phosphate-buffered saline. Pinosylvin (10 mg/kg; Carbosynth) or solvent control (1% dimethyl sulfoxide in saline) was administered 24 h prior to bleomycin exposure and then daily for 14 days until sacrifice. At 14 days after bleomycin administration, the mice were sacrificed, and their lungs were collected for IHC, SA-β-gal staining, and WB. All animal experiments were performed in accordance with the guidelines of the Dankook University Animal Care Committee.

### Statistical analysis

Statistical significance was analyzed using Student’s *t*-test and one-way ANOVA. Statistical differences were determined based on *P* values (*P < 0.05, **P < 0.01, ***P < 0.001).

## Results

### SIRT1 protein expression is downregulated via ubiquitin-mediated proteasomal degradation during SIPS

**Fig. 1:**
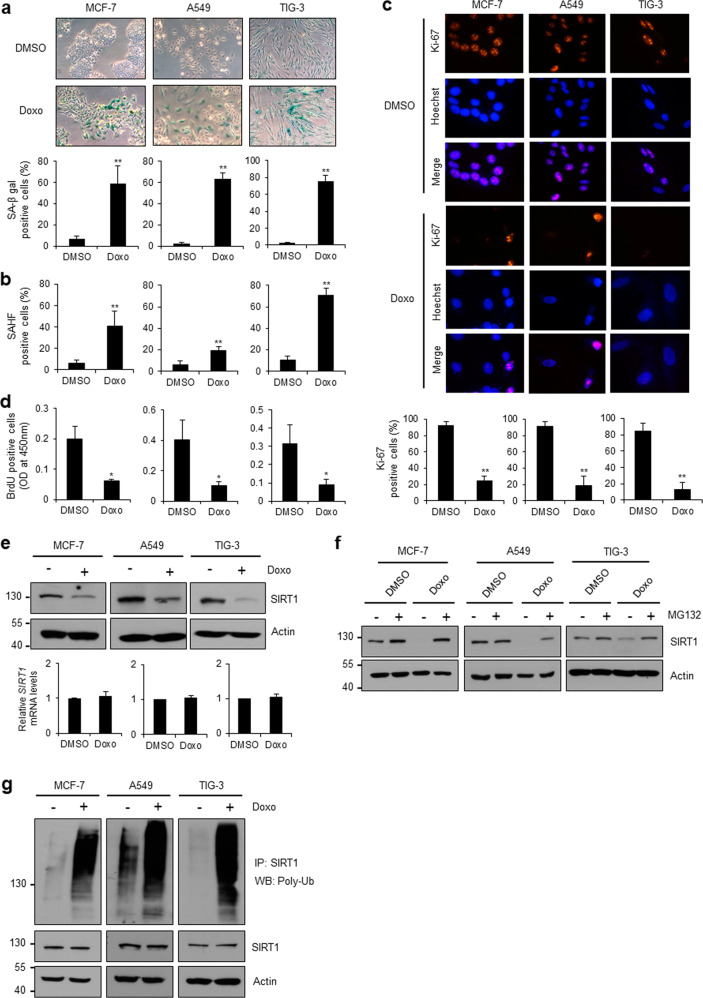
SIRT1 stability is downregulated during DNA damage-induced senescence. **a**–**d** Doxorubicin-induced cellular senescence. Doxorubicin was applied as described in “Materials and methods”. The error bars in all the panels represent the means ± standard deviations (SDs) of three independent experiments. The statistical significance was determined based on *P* values (*P < 0.05, **P < 0.01). **a** Senescence-associated β-galactosidase (SA-β-gal) staining and quantification of SA-β-gal-positive cells. **b** Quantification of senescence-associated heterochromatin foci. Cells were stained with Hoechst 33342 (Sigma‒Aldrich). **c** Ki-67 immunostaining. Cells were immunostained using primary anti-Ki-67 and secondary anti-rhodamine antibodies, and the numbers of Ki-67-positive cells were counted. **d** BrdU incorporation. Cells were incubated with BrdU to measure newly synthesized DNA in proliferating cells. **e** Effect of doxorubicin on SIRT1 protein abundance. The patterns of SIRT1 protein expression were monitored via WB; the mRNA level of SIRT1 was analyzed via RT‒qPCR. **f** Effect of MG132 on doxorubicin-induced SIRT1 downregulation. Doxorubicin-treated cells were incubated with (+) and without (−) 10 μM MG132. **g** Doxorubicin-induced SIRT1 ubiquitination. SIRT1 ubiquitination was visualized via IP using an anti-SIRT1 antibody and WB using an anti-poly-Ub antibody.

Despite its diverse functions, the mechanism controlling the stability of SIRT1 protein remains poorly defined under pathological conditions, including cellular senescence. To determine this mechanism, we first induced premature senescence through DNA damage in MCF-7 breast cancer cells, A549 lung adenocarcinoma cells, and TIG-3 lung fibroblast cells. The cells treated with doxorubicin, a DNA-damaging agent, showed large, flat senescence-related features. Doxorubicin-induced senescence was apparent based on increases in SA-β-gal staining (Fig. 1a) and senescence-associated heterochromatic foci (Fig. 1b) as well as reductions in Ki-67 staining (Fig. 1c) and BrdU incorporation (Fig. 1d). Notably, doxorubicin treatment reduced the level of SIRT1 protein without affecting its mRNA expression pattern (Fig. 1e). Treatment with MG132, a proteasome inhibitor, restored the SIRT1 level in doxorubicin-treated cells (Fig. 1f), indicating the proteasome-dependent degradation of SIRT1. Furthermore, we observed a significant acceleration of SIRT1 polyubiquitination after doxorubicin treatment (Fig. 1g). To extend these findings, we analyzed the stability of SIRT1 under other stress conditions. After overexpression of the activated oncogene *H-RasV12*, we observed SIRT1 degradation and ubiquitination along with senescence characteristics in both MCF-7 and TIG-3 cells (Supplementary Fig. 1a–d). Similar results were obtained with A549 and TIG-3 cells that had been treated with H_2_O_2_, which causes oxidative stress (Supplementary Fig. 1e–h). Collectively, these results suggest that SIRT1 protein expression is downregulated via ubiquitin-mediated proteasomal degradation under various stress-induced senescent conditions.

### APC/C-Cdh1 promotes the ubiquitin-dependent proteasomal degradation of SIRT1

**Fig. 2:**
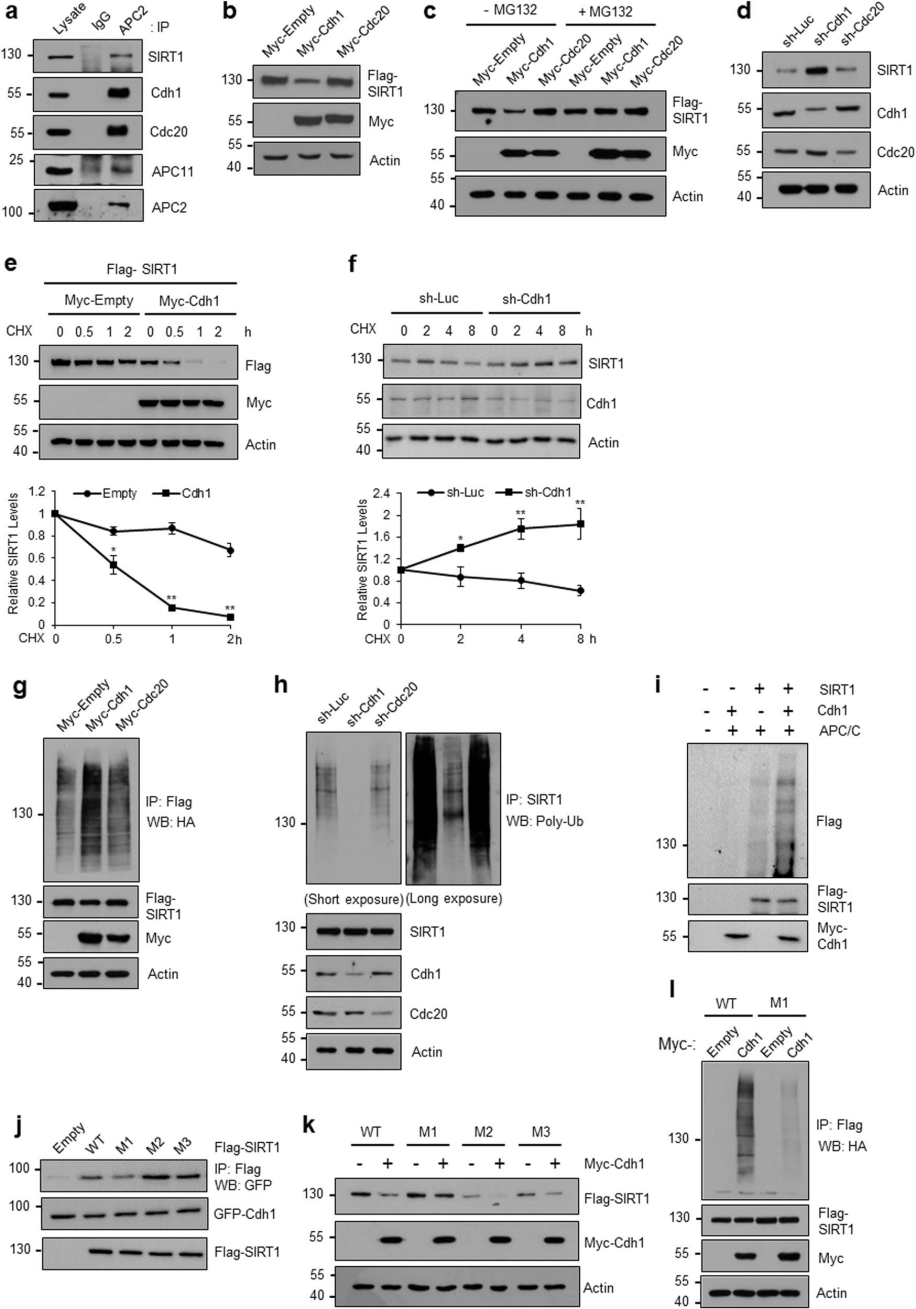
APC/C-Cdh1 promotes SIRT1 degradation and ubiquitination. **a** SIRT1 interacts with the APC/C complex. HEK293 cell lysates were prepared for IP using an anti-IgG antibody or anti-APC2 antibody. Precipitated proteins were detected via WB using the indicated antibodies. **b** Cdh1 induces SIRT1 degradation. Either Myc-Cdh1 or Myc-Cdc20 was coexpressed with Flag-SIRT1 in HEK293 cells. Cell lysates were analyzed via WB using individual antibodies. **c** The Cdh1-mediated degradation of SIRT1 was restored by the proteasome inhibitor MG132. **d** Cdh1 knockdown increases the SIRT1 levels. HEK293 cells were transfected with sh-Luciferase (Luc) as a control, sh-Cdh1, or sh-Cdc20. After 48 h, the cells were harvested, and WB was conducted with the indicated antibodies. **e**, **f** Effects of Cdh1 overexpression (**e**) and depletion (**f**) on the turnover rate of SIRT1. HEK293 cells were transfected as indicated and treated with 0.1 μM doxorubicin for 1 h. One day after doxorubicin withdrawal, the cells were treated with CHX (50 μg/ml) for the indicated times and then subjected to WB for the indicated proteins. The relative protein level of SIRT1 was quantified using ImageJ software. The error bars represent the means ± SDs of three independent experiments (*P < 0.05, **P < 0.01). **g**, **h** Effects of Cdh1 overexpression (**g**) and depletion (**h**) on SIRT1 ubiquitination. Twenty-four hours after the transfection of HEK293 cells under the indicated conditions, the cells were treated with 10 μM MG132 for 12 h, harvested, and subjected to IP using an anti-Flag antibody. SIRT1 ubiquitination was visualized via WB using an anti-HA antibody or anti-poly-Ub antibody. **i** In vitro ubiquitination of SIRT1 by Cdh1. In vitro-translated Flag-SIRT1 and Myc-Cdh1 were incubated with a ubiquitination kit (Enzo Life Science), and APC/C complexes were immunopurified using an anti-APC2 antibody. Ubiquitinated SIRT1 was detected via WB using an anti-Flag antibody. **j**–**l** Identification of the SIRT1 D-box needed for Cdh1 targeting. **j** Impaired interaction between Cdh1 and the SIRT1 M1 mutant. HEK293 cells were cotransfected as indicated. Cell lysates were subjected to IP using an anti-Flag antibody and WB using an anti-GFP antibody. **k** The SIRT1 M1 mutant is resistant to Cdh1-induced degradation. HEK293 cells were transfected as indicated. WB was conducted using the indicated antibodies. **l** Defective SIRT1 ubiquitination with M1 mutation. HEK293 cells were transfected with the indicated expression vectors, including HA-ubiquitin. Ubiquitinated SIRT1 was visualized via IP using an anti-Flag antibody, followed by WB using an anti-HA antibody.

The APC/C coactivator Cdh1 has a critical role in DNA damage-induced premature cellular senescence^20–22^. Several studies have shown that SIRT1 also antagonizes senescence induced by DNA damage, cigarette smoke, and insulin-like growth factor-1^35–37^. Based on these findings, we hypothesized that DNA damage-triggered Cdh1 activation could lead to SIRT1 degradation and subsequent induction of premature senescence. Notably, the downregulation of SIRT1 expression in doxorubicin-induced senescent cells coincided with reductions in the protein levels of Cdc20 and Cyclin B1, which are substrates of Cdh1 (Supplementary Fig. 2-1a). Similar results were obtained with oncogenic *Ras*-expressing cells (Supplementary Fig. 2-1b). These data prompted us to investigate whether SIRT1 physically interacts with Cdh1. As demonstrated by IP using an anti-APC2 antibody and subsequent WB analysis, SIRT1 interacts with APC2 in vivo (Fig. 2a) in a manner similar to that found for APC11 and the APC/C coactivators Cdh1 and Cdc20. Further IP using an anti-Myc antibody (Myc-Cdh1) under overexpression conditions confirmed the interaction between Cdh1 and SIRT1 (Supplementary Fig. 2-2a). Intriguingly, the SIRT1 protein levels decreased after the addition of Cdh1 but not the addition of Cdc20 (Fig. 2b). MG132 treatment restored the level of SIRT1 protein in Cdh1-overexpressing cells (Fig. 2c), suggesting the proteasomal dependence of SIRT1 degradation. Importantly, Cdh1 depletion using shRNA led to increases in the endogenous SIRT1 levels (Fig. 2d). Using CHX, an inhibitor of protein synthesis, we measured the turnover rate of SIRT1 protein. Cdh1 overexpression substantially decreased the half-life of Flag-tagged SIRT1 (Fig. 2e), and this effect was reversed upon Cdh1 knockdown (Fig. 2f), confirming that Cdh1 facilitates the turnover of SIRT1 protein. We next addressed whether Cdh1 promotes SIRT1 degradation via direct polyubiquitination. The coexpression of Cdh1 with SIRT1 significantly increased SIRT1 ubiquitination in vivo, whereas Cdc20 did not affect SIRT1 ubiquitination (Fig. 2g). Conversely, Cdh1 suppression induced a significant reduction in the ubiquitination of endogenous SIRT1 (Fig. 2h). To further explore whether the effect of Cdh1 is direct, we performed ubiquitination assays using Cdh1 and SIRT1 proteins that had been synthesized in vitro. SIRT1 was extensively ubiquitinated upon mixing with Cdh1 in the presence of the immunopurified APC/C complex (Fig. 2i). Taken together, these findings suggest that the APC/C coactivator Cdh1 directly mediates SIRT1 polyubiquitination, which presumably reflects the underlying mechanism of SIRT1 protein downregulation during stress-induced senescence.

Cdh1 and Cdc20 specifically bind and recruit substrates to the APC/C complex via recognition of D-box or KEN-box destruction motifs^13,14,38^. We found three putative D-box motifs (RXXL) in the amino acid sequence of SIRT1, which were highly conserved among species (Supplementary Fig. 2-2b). To determine which SIRT1 D-boxes are necessary for Cdh1 recognition, we constructed point mutants of SIRT1 in which arginine (R) and leucine (L) residues in the D-box were replaced with alanine (AXXA); these mutants were designated SIRT1 M1, M2, and M3. Co-IP analysis indicated that the binding of Cdh1 to SIRT1 M1 was weaker than its binding to wild-type (WT) SIRT1 and the other mutants (Fig. 2j). As expected, SIRT1 M1 was more resistant to Cdh1-mediated degradation than the WT or other mutants of SIRT1 (Fig. 2k, Supplementary Fig. 2-2c). Consistent with this observation, the Cdh1-promoted ubiquitination of SIRT1 M1 was significantly reduced in vivo (Fig. 2l), supporting the requirement of the first D-box of SIRT1 for Cdh1-mediated ubiquitination.

### Cdh1 is required for SIRT1 degradation and stress-induced senescence

**Fig. 3:**
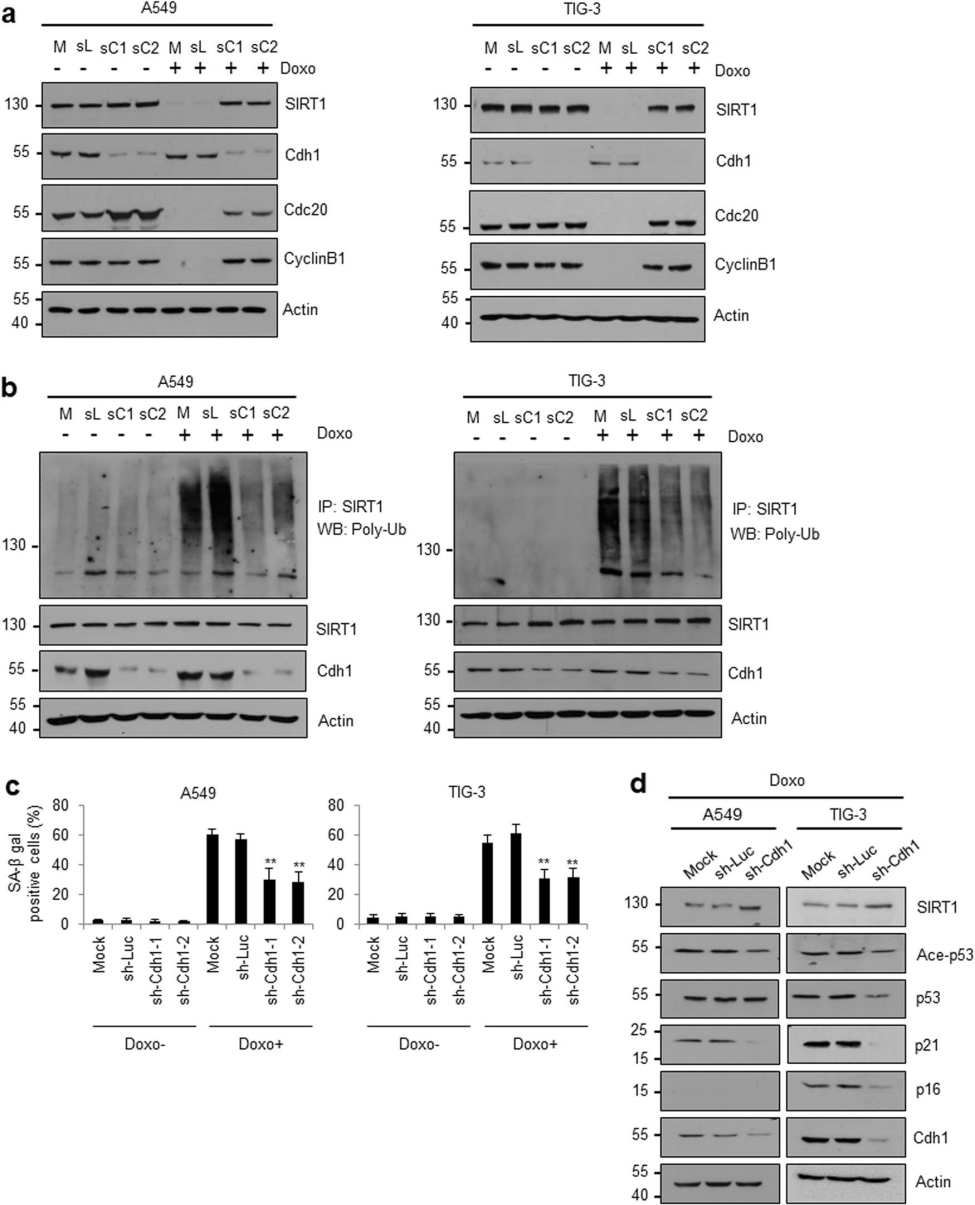
Cdh1 depletion impairs SIRT1 ubiquitination and DNA damage-induced senescence. Effects of Cdh1 knockdown on SIRT1 degradation (**a**), SIRT1 ubiquitination (**b**), cellular senescence (**c**), and expression patterns of senescence-associated proteins (**d**). A549 or TIG-3 cells were transfected with two types of sh-Cdh1 (sC1 and sC2) or control sh-Luc (sL) or mock transfected (M) before doxorubicin treatment. **a** Protein expression patterns were monitored via WB using the indicated antibodies. **b** Cells were additionally treated with 10 μM MG132 for 12 h before harvest. SIRT1 ubiquitination was examined via IP using an anti-SIRT1 antibody, followed by WB using an anti-poly-Ub antibody. **c** After transfection and doxorubicin treatment, the numbers of SA-β-gal-positive cells were quantified. The error bars represent the means ± SDs of three independent experiments (**P < 0.01). **d** The protein expression patterns were monitored via WB using the indicated antibodies. A549 cells are deficient in p16.

Considering the downregulation of SIRT1 protein expression under stress and the key role of Cdh1 in SIRT1 ubiquitination, we hypothesized that Cdh1 is responsible for SIRT1 turnover during stress-induced senescence. To test this hypothesis, we explored the effect of Cdh1 silencing on the SIRT1 protein expression pattern in doxorubicin-induced senescent cells. After Cdh1 knockdown, SIRT1 degradation was significantly abrogated in both A549 and TIG-3 cells (Fig. 3a). The Cdh1 substrates Cdc20 and CyclinB1 were restored by Cdh1 knockdown in these cells. Additionally, loss of Cdh1 decreased the ubiquitination of SIRT1 (Fig. 3b) and the abundance of senescent cells in the presence of doxorubicin (Fig. 3c). Consistent with this observation, similar effects of Cdh1 knockdown were observed in oncogenic *Ras*-induced senescent cells: SIRT1 degradation, SIRT1 ubiquitination, and SA-β-gal staining (Supplementary Fig. 3a–c). Finally, we measured the effect of Cdh1 depletion on the expression patterns of proteins involved in the SIRT1-p53 signaling pathway in doxorubicin-treated cells. We observed apparent upregulation of SIRT1 in Cdh1-depleted cells accompanied by reduced p53 acetylation, which led to suppression of p21. The expression of p16, a senescence marker, was effectively impaired by Cdh1 knockdown in TIG-3 cells but not in p16-deficient A549 cells (Fig. 3d). Overall, our data suggest that Cdh1, a coactivator of the E3 ubiquitin ligase APC/C, mediates ubiquitin-dependent SIRT1 degradation during stress-induced cellular senescence.

### AROS attenuates DNA damage-induced senescence by stabilizing the SIRT1 protein

**Fig. 4:**
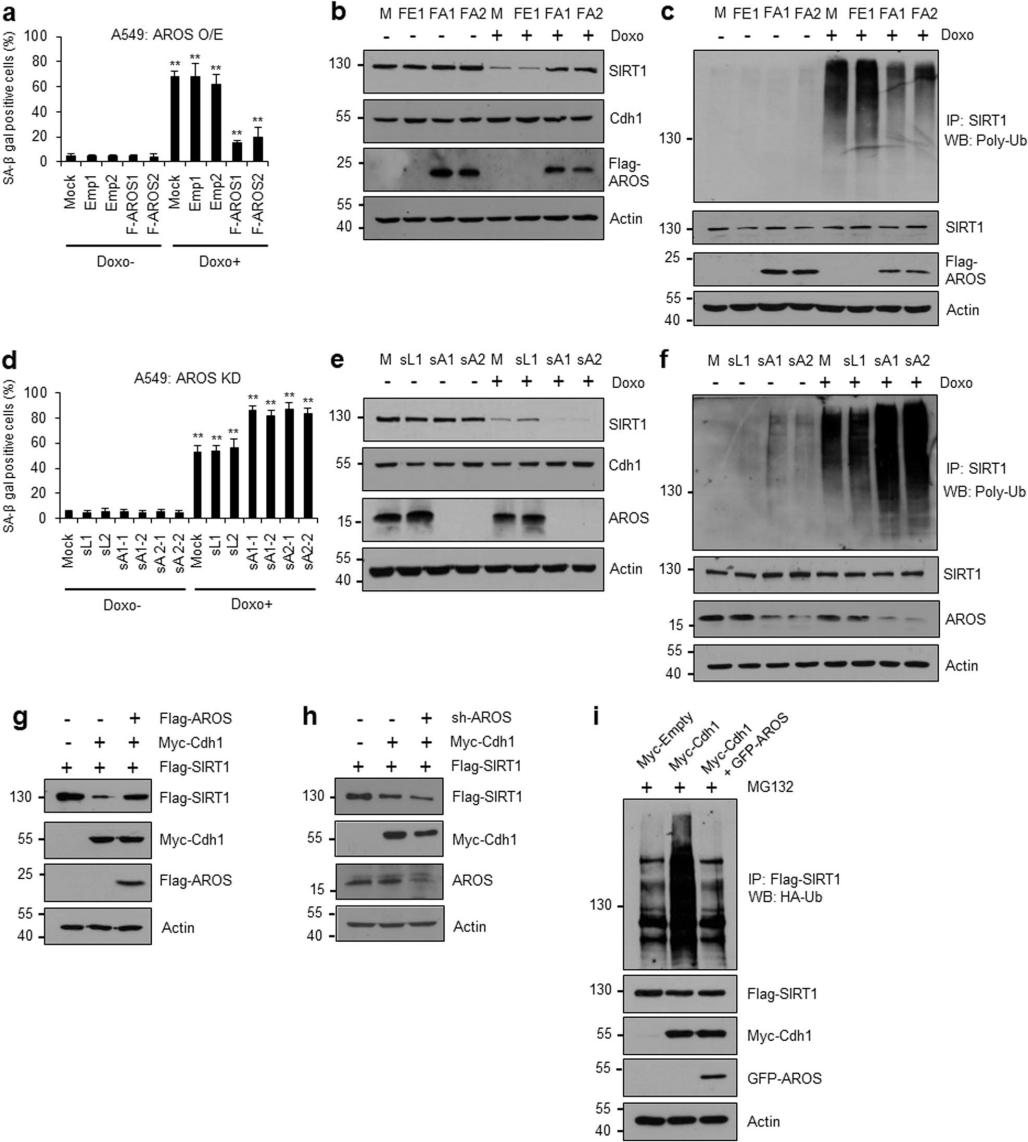
AROS abrogates DNA damage-induced senescence by stabilizing SIRT1. **a**–**c** Effects of AROS overexpression on doxorubicin-induced senescence (**a**), SIRT1 degradation (**b**), and SIRT1 ubiquitination (**c**). Doxorubicin was applied to two subclones of A549 cells (F-AROS1 and F-AROS2) that stably express Flag-AROS. **a** After 4 days, the cells were examined via SA-β-gal staining. The error bars represent the means ± SDs of three independent experiments (**P < 0.01). **b** Under the same conditions, the SIRT1 levels were monitored via WB using the indicated antibodies. **c** After the addition of MG-132, SIRT1 ubiquitination was measured via IP using an anti-SIRT1 antibody and WB using an anti-poly-Ub antibody. **d**–**f** Effects of AROS knockdown on doxorubicin-induced senescence (**d**), SIRT1 degradation (**e**), and SIRT1 ubiquitination (**f**). AROS-depleted A549 cells were selected using two types of sh expression vectors. Two subclones of AROS-depleted A549 cells were treated with (+) or without (−) doxorubicin. **d** The numbers of SA-β-gal-positive cells were counted. The error bars represent the means ± SDs of three independent experiments (**P < 0.01). **e** SIRT1 degradation was monitored via WB using the indicated antibodies. **f** SIRT1 ubiquitination was monitored as described above. **g**, **h** Effects of ectopic AROS overexpression (**g**) and knockdown (**h**) on SIRT1 stabilization. HEK293 cells were transiently transfected with Flag-SIRT1, Myc-Cdh1, and Flag-AROS (for overexpression) or sh-AROS (for knockdown). Cell lysates were subjected to WB using the antibodies shown on the right. **i** Effect of AROS overexpression on Cdh1-promoted SIRT1 ubiquitination. HEK293 cells were transfected as indicated in the presence of MG-132. Ubiquitinated SIRT1 was visualized via IP using an anti-Flag antibody and WB using an anti-HA antibody. M, mock; FE1, Flag-empty #1; FA1, Flag-AROS #1; FA2, Flag-AROS #2; sL1, sh-Luc #1; sL2, sh-Luc #2; sA1-1, sh-AROS #1-1; sA1-2, sh-AROS #1-2; sA2-1, sh-AROS #2-1; and sA2-2, sh-AROS #2-2.

In a previous study, we identified AROS as an endogenous SIRT1 activator that facilitates SIRT1 deacetylase activity^32^. However, the mechanism by which AROS regulates SIRT1 activity and its role in cellular senescence has remained unclear. In this study, we aimed to determine the role of AROS in doxorubicin-induced senescence. Surprisingly, the number of SA-β-gal-positive cells was significantly reduced in doxorubicin-treated AROS-expressing cells compared to controls (Fig. 4a, Supplementary Fig. 4a). The coexpression of AROS restored the level of SIRT1 protein, which was downregulated after doxorubicin treatment, and did not affect the level of Cdh1 protein (Fig. 4b) or pattern of SIRT1 mRNA expression (Supplementary Fig. 4b). Similarly, AROS impaired ubiquitin conjugation to SIRT1 (Fig. 4c). Conversely, the knockdown of endogenous AROS increased the proportion of SA-β-gal-positive cells among doxorubicin-treated cells (Fig. 4d, Supplementary Fig. 4c). Moreover, AROS depletion accelerated the doxorubicin-induced degradation of SIRT1 (Fig. 4e) and SIRT1 polyubiquitination (Fig. 4f). We next explored whether the effect of AROS is related to the regulation of SIRT1 by Cdh1 under general conditions. The coexpression of AROS with Cdh1 and SIRT1 restored the level of SIRT1 that had been decreased by Cdh1 (Fig. 4g). This observation was reversed by the silencing of AROS, and AROS knockdown accelerated the Cdh1-promoted degradation of SIRT1 (Fig. 4h). Furthermore, AROS reduced the SIRT1 ubiquitination level to normal levels after it was elevated by Cdh1 overexpression (Fig. 4i). In summary, these results suggest that AROS inhibits Cdh1-mediated SIRT1 ubiquitination and thus stabilizes the SIRT1 protein levels, supporting a potential role for AROS in stress-induced senescence. Intriguingly, we measured the effect of AROS and Cdh1 on SIRT1 turnover under different DNA damage conditions using the DNA topoisomerase inhibitors etoposide and camptothecin. As observed using doxorubicin, this experiment yielded similar data (Supplementary Figs. 5-1 and 5-2), which suggested that the opposite roles of AROS and Cdh1 in SIRT1 degradation are conserved under various DNA-damaging- and senescence-inducing conditions.

### AROS competes with Cdh1 for binding and stabilizing SIRT1

**Fig. 5:**
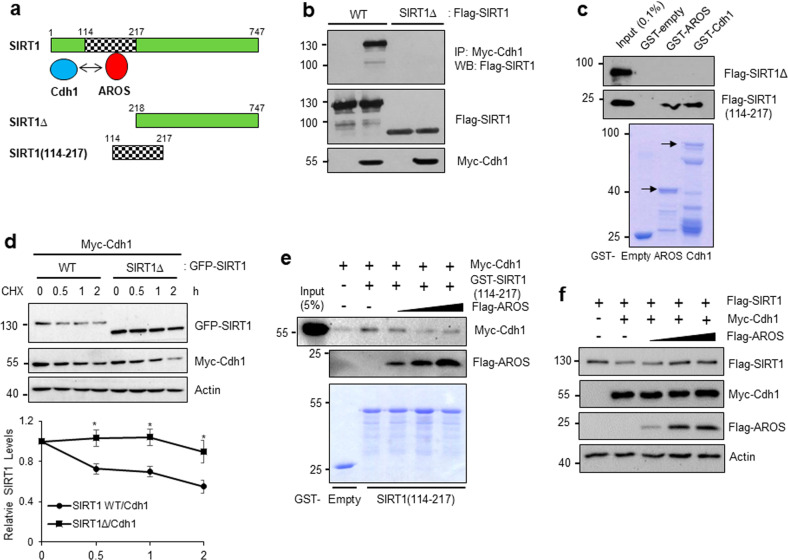
AROS competes with Cdh1 to bind and stabilize SIRT1. **a** Schematic diagram of competition between AROS and Cdh1 for SIRT1 binding. Amino acids 114–217 in SIRT1 comprise the AROS-binding site. SIRT1∆ represents the deletion of amino acids 1–217 of SIRT1. **b** Requirement of amino acids 1–217 of SIRT1 for Cdh1 binding. HEK293 cells were transfected with Myc-Cdh1 and Flag-SIRT1 WT or Flag-SIRT1∆. Cell lysates were subjected to IP using an anti-Myc antibody and WB using an anti-Flag antibody. **c** Direct interaction of the SIRT1 fragment with AROS and Cdh1. Purified GST, GST-AROS, or GST-Cdh1 protein was incubated with in vitro-translated Flag-SIRT1∆ or the Flag-SIRT1(114–217) fragment. The bound proteins were visualized via WB using an anti-Flag antibody. **d** Requirement of amino acids 1–217 of SIRT1 for Cdh1-mediated SIRT1 turnover. HCT116 cells were transiently transfected with Myc-Cdh1 and GFP-SIRT1-WT or GFP-SIRT1∆ and further treated with CHX (50 μg/ml) for the indicated time periods. Images were visualized by WB using individual antibodies. The error bars represent the means ± SDs of three independent experiments (*P < 0.05). **e** Competition for SIRT1 binding. GST or GST-SIRT1(114–217) and in vitro-translated Myc-Cdh1 were incubated with increasing amounts of in vitro-translated Flag-AROS. The bound proteins were visualized via WB using the indicated antibodies. **f** Competition for SIRT1 stability. HEK293 cells were transfected with Flag-SIRT1, Myc-Cdh1, and increasing amounts of Flag-AROS. The protein levels were monitored by WB using the indicated antibodies.

Next, we investigated the molecular mechanism by which AROS suppresses Cdh1-promoted SIRT1 ubiquitination. Notably, we realized that two SIRT1 D-box motifs (M1 and M2) are located within the AROS-binding region (amino acids 114–217)^32^. Therefore, we hypothesized that AROS directly competes with Cdh1 for SIRT1 binding and thereby disrupts Cdh1-mediated SIRT1 ubiquitination (Fig. 5a). To test this hypothesis, we performed a co-IP assay with Cdh1 using WT SIRT1 and a SIRT1∆ (amino acids 218–747) construct that lacks both the AROS-binding region and two D-box motifs. As predicted, no apparent interaction between Cdh1 and SIRT1∆ was observed, whereas an interaction was found with WT SIRT1 (Fig. 5b). GST pull-down assays revealed that both AROS and Cdh1 directly interact with the AROS-binding region of SIRT1, but not with SIRT1∆ (Fig. 5c). Cdh1-induced SIRT1 degradation was impaired by the deletion of SIRT1 amino acids 218–747 (Fig. 5d), suggesting that Cdh1 binding is necessary for SIRT1 destabilization. To further characterize the roles of AROS and Cdh1 in SIRT1 binding, we performed competitive binding experiments in vitro. The addition of increasing amounts of AROS led to corresponding increases in the disruption of the interaction between Cdh1 and SIRT1(114–217) (Fig. 5e). Consistent with this finding, Cdh1-mediated SIRT1 protein downregulation was gradually rescued by increases in AROS expression (Fig. 5f). Overall, these results indicate that AROS disrupts Cdh1–SIRT1 interactions via competitive association with SIRT1 and impedes Cdh1-mediated SIRT1 ubiquitination, which results in stabilization of the SIRT1 protein levels.

### Cdh1 and AROS have opposing roles in the epigenetic regulation of SASP-associated genes

**Fig. 6:**
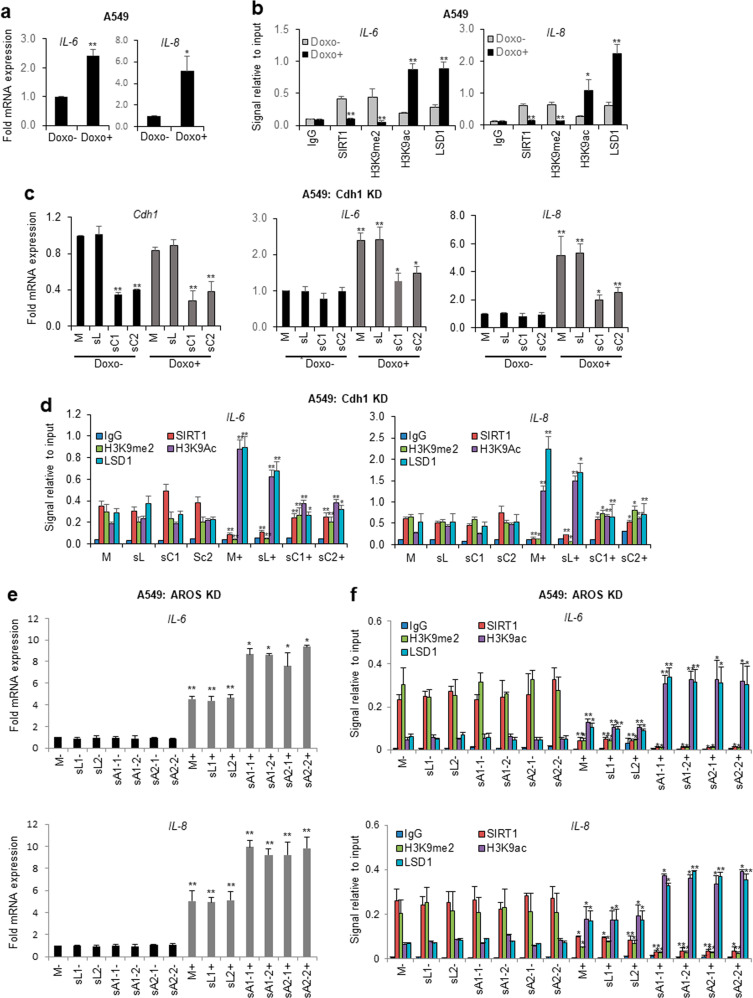
Cdh1 and AROS have opposing roles in the epigenetic regulation of SASP-associated genes. **a** Upregulation of *IL-6* and *IL-8* mRNA expression levels by doxorubicin. The mRNA expression levels in doxorubicin-treated A549 cells were measured via RT‒qPCR and normalized to GAPDH expression. **b** Effect of doxorubicin on the epigenetic regulation of SASP genes. A549 cells were treated with doxorubicin and subjected to quantitative chromatin IP (qChIP) analysis using the indicated antibodies. **c** Effect of Cdh1 depletion on *IL-6* and *IL-8* mRNA expression patterns. A549 cells were transfected with sh-Luc (sL) or sh-Cdh1 (sC) and then treated with doxorubicin. The mRNA level was quantified via RT‒qPCR. **d** Effect of Cdh1 knockdown on the epigenetic regulation of SASP genes. A549 cells were transfected and treated as described above and then subjected to qChIP assays of the *IL-6* and *IL-8* promoters using the indicated antibodies. **e** Effect of AROS depletion on *IL-6* and *IL-8* mRNA expression patterns. A549 cells with stable AROS knockdown were treated with doxorubicin and then subjected to RT‒qPCR analysis. **f** Opposing effects of AROS knockdown on the epigenetic regulation of SASP genes. A549 cells with stable AROS knockdown and with (+) and without (−) doxorubicin treatment were subjected to qChIP analysis using the indicated antibodies. M, Mock; sL, sh-Luc; sC1, sh-Cdh1 #1; sC2, sh-Cdh1 #2; sL1, sh-Luc #1; sL2, sh-Luc #2; sA1-1, sh-AROS #1-1; sA1-2, sh-AROS #1-2; sA2-1, sh-AROS #2-1; and sA2-2, sh-AROS #2-2. The error bars in all the panels represent the means ± SDs of three independent experiments (*P < 0.05, **P < 0.01).

Cellular senescence induces SASP, which is associated with the regulation of inflammation and alteration of the surrounding microenvironment^4,5^. Recent studies have noted that the expression levels of two major SASP components, IL-6 and IL-8, are epigenetically upregulated by the Cdh1-mediated degradation of G9a, a major H3K9 methyltransferase; these changes lead to reduced enrichment of the H3K9me1/me2 levels in response to DNA damage^20^. The expression levels of IL-6 and IL-8 are also upregulated via the SIRT1 downregulation-driven acetylation of histone H3K9 and H4K16 in senescent cells^39^. Moreover, we previously reported that SIRT1 and LSD1 oppositely regulate the epigenetic suppression of PPARγ activity^40^. Therefore, based on these references, we hypothesized that Cdh1 and AROS, together with SIRT1, might have opposing roles in the regulation of SASP-related genes during stress-induced senescence. To test this hypothesis, we analyzed the effect of doxorubicin on the expression patterns of two SASP-associated genes, *IL-6* and *IL-8*. RT‒qPCR showed that the treatment of A549 cells with doxorubicin led to significantly increased mRNA expression levels of both genes (Fig. 6a). Subsequent ChIP assays demonstrated reductions in the recruitment of SIRT1 to the promoters, which were likely related to the downregulation of SIRT1 protein expression under DNA damage conditions, and these reductions led to the enrichment of H3K9ac (active histone marker) (Fig. 6b). Furthermore, we observed reduced deposition of the repressive histone marker H3K9me2, which coincided with increased recruitment of the histone demethylase LSD1, and these effects led to reduced H3K9me2 levels and activation of the *IL-6* and *IL-8* genes in doxorubicin-treated A549 cells. To assess the role of Cdh1 in the epigenetic regulation of SASP genes, we performed Cdh1 depletion (Fig. 6c, left). Cdh1 silencing reversed the effect of doxorubicin on gene expression relative to the controls, presumably by stabilizing SIRT1 (Fig. 6c, middle and right). The effects of doxorubicin on the binding capacities of SIRT1 and LSD1 and on the deposition of H3K9me2 and H3K9ac on gene promoters were completely reversed by Cdh1 knockdown (Fig. 6d). To exclude cell-type specific effects, we used other cell lines, TIG-3 (Supplementary Fig. 6a–d) and MCF-7 (data not shown), to confirm the role of Cdh1 in the epigenetic control of SASP-related genes. We then assessed whether AROS affects the expression of *IL-6* and *IL-8* in doxorubicin-induced senescent cells. Notably, AROS knockdown led to the enhanced doxorubicin-promoted expression of *IL-6* and *IL-8* genes in A549 cells compared to controls (Fig. 6e). No apparent effect of AROS silencing was observed in the absence of doxorubicin. However, significant downregulation of SIRT1 and H3K9me2 and upregulation of LSD1 and H3K9ac were observed in doxorubicin-treated cells. This effect of doxorubicin was accelerated in AROS-depleted senescent cells (Fig. 6f). Notably, these changes were reversed by ectopic expression of AROS (Supplementary Fig. 6e, f). These findings indicate that the effect of doxorubicin is reversed in Cdh1-depleted senescent cells and accelerated in AROS-depleted senescent cells. Overall, we concluded that Cdh1 and AROS have opposing roles in the epigenetic control of SASP gene expression via modulation of chromatin association with SIRT1 and modified histones (H3K9me2 and H3K9ac), likely due to opposing regulation of SIRT1 stability during stress-induced cellular senescence.

### Pinosylvin inhibits bleomycin-induced pulmonary senescence by stabilizing SIRT1

**Fig. 7:**
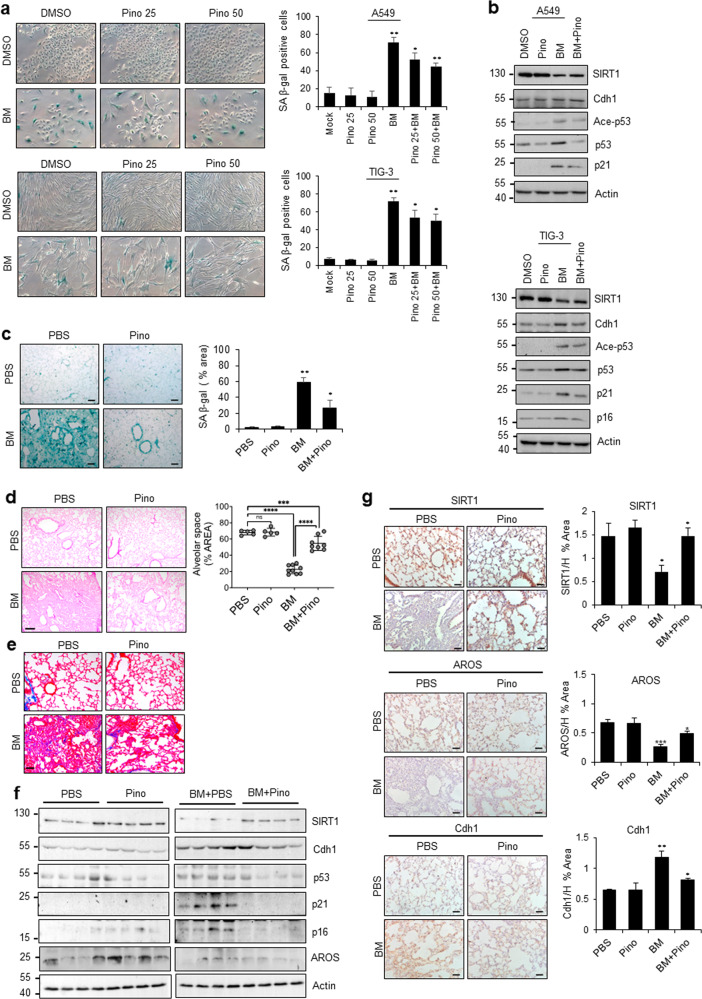
The reciprocal expression patterns of AROS and Cdh1 are associated with pinosylvin-mediated SIRT1 stabilization in bleomycin-induced pulmonary senescence. **a** Inhibition of bleomycin-induced cellular senescence by pinosylvin. A549 or TIG-3 cells were treated with 25 μM or 50 μM pinosylvin prior to exposure to bleomycin. A549 and TIG-3 cells were treated with bleomycin at concentrations of 5 μM and 2.5 μM, respectively, for 24 h. Bleomycin-induced senescence was monitored via microscopy and quantified by counting the number of SA-β-gal-positive cells. The error bars represent the means ± SDs of three independent experiments (*P < 0.05, **P < 0.01). **b** Effect of pinosylvin on the p53/p21 senescence pathway in bleomycin-induced senescent cells. The protein expression patterns were examined via WB using the antibodies shown on the right. **c** Protection against bleomycin-induced mouse pulmonary senescence by pinosylvin in vivo. Mice were treated as described in “Materials and methods”. The level of SA-β-gal staining was analyzed using ImageJ software. Scale bar, 20 μm. **d** Effect of pinosylvin on the histological and morphometric patterns. Hematoxylin and eosin (H&E) staining and morphometric analysis of the alveolar space in PBS (*n* = 5), Pino (*n* = 5), BM (*n* = 9), and BM/Pino (*n* = 8)-administered lung sections. **e** Masson’s trichrome staining of PBS-, Pino-, BM-, and BM/Pino-administered lung sections. The error bars show the means ± S.E.Ms.; ns, not significant; ***P < 0.001 and ****P < 0.0001, two-tailed Student’s *t*-test. Scale bars: 200 μm (**d**) and 100 μm (**e**). **f** Effect of pinosylvin on the expression patterns of genes associated with the p53/p21 and p16 pathways. The protein expression patterns were determined via WB using the indicated antibodies. **g** Effect of pinosylvin on the expression patterns of AROS and Cdh1. After combined treatment with pinosylvin and bleomycin, mouse lung tissues were subjected to IHC staining using antibodies against SIRT1, AROS, and Cdh1. PBS (*n* = 4–5), Pino, BM, and BM/Pino (*n* = 5). The stained images were analyzed using ImageJ software. Scale bar, 20 μm. The error bars represent the means ± SDs of experiments using four mice (*P < 0.05, **P < 0.01, ***P < 0.001).

Cellular senescence is a major cause of fibrotic pulmonary disease^41,42^. SIRT1 has been reported to negatively regulate pulmonary fibrosis^43,44^. Thus, it is necessary to determine whether SIRT1, Cdh1, and AROS are functionally associated with pulmonary fibrosis. Pinosylvin, a natural stilbenoid polyphenol that is structurally similar to resveratrol, was recently characterized as a natural activator of SIRT1 in skeletal muscle cells^45^ and adipocytes^46^. To determine whether pinosylvin influences stress-induced senescence, we used bleomycin, which has been reported to induce pulmonary senescence^47^. As expected, bleomycin treatment strongly induced senescence as measured by SA-β-gal staining, whereas prior treatment with pinosylvin reduced bleomycin-induced SA-β-gal activity in A549 and TIG-3 cells (Fig. 7a). An antisenescence effect of pinosylvin was observed in A549 and TIG-3 cells that had been treated with doxorubicin (Supplementary Fig. 7a). To elucidate the underlying mechanism, cells were treated with pinosylvin, bleomycin, or both and then subjected to WB analysis. Bleomycin treatment resulted in SIRT1 protein downregulation, leading to p53 acetylation and p21 induction, which are indicators of senescence (Fig. 7b). Upregulation of p16 expression was observed only in TIG-3 cells due to p16 deficiency in A549 cells. The addition of pinosylvin increased the SIRT1 protein level compared with that observed with bleomycin treatment alone, and this effect was accompanied by p53 deacetylation and p21 downregulation in both cell types (Fig. 7b). Similar results were obtained with doxorubicin treatment (Supplementary Fig. 7b). Next, to investigate the protective effect of pinosylvin on the regulation of pulmonary senescence in vivo, we established an experimental system using bleomycin-induced pulmonary fibrosis in mice^48^. To induce pulmonary fibrosis, mice were treated with a single intratracheal dose of bleomycin. Pinosylvin was administered 24 h prior to bleomycin exposure and daily thereafter. As determined by SA-β-gal staining, bleomycin-induced pulmonary senescence was greatly impaired by pinosylvin treatment (Fig. 7c). In addition, the histological characteristics of pulmonary fibrosis were determined by H&E staining and Masson’s trichrome staining. H&E staining indicated obvious histological changes and destruction of the lung structure after bleomycin treatment, and these results were rescued by pinosylvin treatment (Fig. 7d). Similar results were obtained by Masson’s trichrome staining (Fig. 7e). Consistent with these results, we observed increased expression levels of the fibrosis markers alpha-smooth muscle actin (α-SMA), collagen I, fibronectin and elastin after bleomycin treatment. In contrast, the addition of pinosylvin significantly reduced the levels of these proteins (Supplementary Fig. 7c*–*e).

We next explored whether pinosylvin affects the p53/p21 pathway and p16 expression patterns during bleomycin-induced pulmonary senescence. After bleomycin treatment, the expression levels of p53, p21, and p16 were highly elevated, and this effect was accompanied by SIRT1 downregulation. These effects were reversed by the addition of pinosylvin (Fig. 7f). Notably, Cdh1 expression increased after bleomycin treatment and decreased after pinosylvin addition, which resulted in the AROS levels remaining unchanged. Furthermore, an IHC analysis of lung tissues revealed that the expression levels of SIRT1 and AROS proteins were downregulated after bleomycin treatment and restored by pinosylvin treatment, whereas Cdh1 showed the opposite expression pattern (Fig. 7g), indicating a potential role of pinosylvin in SIRT1 stabilization and activation. Taken together, these results indicate that pinosylvin prevents bleomycin-induced pulmonary senescence by increasing the levels of SIRT1 and its activator AROS while decreasing that of Cdh1, a SIRT1 destabilizer.

## Discussion

Cellular senescence occurs in response to various stressors and is considered a major contributor to organismal aging. SIRT1 is a well-conserved NAD^+^-dependent deacetylase that cleaves acetyl groups from histones H3 and H4 as well as nonhistone proteins (e.g., p53 and FoxO). Because SIRT1 has pivotal roles in the regulation of aging-related diseases and cellular senescence, research has focused on senescence pathways and the identification of targets regulated by SIRT1; the mechanism underlying the control of SIRT1 protein stability during cellular senescence remains poorly defined. In the present study, we demonstrated that SIRT1 ubiquitination is promoted by APC/C-Cdh1 and followed by proteasomal degradation during stress-induced cellular senescence. The loss of SIRT1 under stress conditions is restored by Cdh1 depletion and accompanied by reduced cellular senescence, which suggests a critical role for Cdh1 in stress-induced SIRT1 degradation in senescent cells. SIRT1 may be ubiquitinated by the E3 ligase MDM2 in response to DNA damage^27^, by CHFR under oxidative stress^28^, by SMURF2 to suppress cell proliferation and tumor formation^49^, and by Grail to promote hepatic steatosis^50^. However, no association with cellular senescence has been observed in previous studies. Notably, other studies have indicated that SIRT1 undergoes autophagosome-lysosomal degradation rather than ubiquitin-dependent proteasomal degradation during DNA damage-induced cellular senescence and aging in vivo^29^. We propose that SIRT1 is a cell cycle-independent substrate for Cdh1, a coactivator of the E3 ubiquitin ligase APC/C, which mediates SIRT1 protein downregulation during stress-induced cellular senescence.

APC/C is a master regulator of the cell cycle that promotes anaphase and mitotic exit and functions as an E3 ubiquitin ligase that ubiquitinates key factors for proteasomal degradation (e.g., cyclin B, securin, Plk1, and Aurora A and B)^12–14^. The enzymatic activity and substrate specificity of APC/C are regulated through associations with the coactivators Cdh1 and Cdc20, which recruit substrates and cause conformational changes to support cooperation with E2 ubiquitin-conjugating enzymes^16^. In addition to cell cycle regulation, recent studies have documented cell cycle-independent functions of APC/C, including the binding of diverse substrates specific to either Cdh1 or Cdc20^51^. One member of the sirtuin family, SIRT2, deacetylates both Cdh1 and Cdc20, supporting mitosis and genome integrity^52^. Recently, SIRT6 was reported to cooperate with APC/C to drive mitosis through the deacetylation of Cdh1, and this protein also serves as a substrate for APC/C^53^. In cell cycle control, SIRT1 promotes cell cycle progression by deacetylating the cell cycle checkpoint kinase CHX2, which leads to its dephosphorylation and inactivation^54^. SIRT1 also deacetylates the centrosome protein polo-like kinase 2 and promotes its ubiquitin-dependent degradation, thereby blocking centriole duplication^55^. However, the functions of SIRT1 in cell cycle progression, including mitosis, remain largely unclear. Considering our finding that SIRT1 is a specific substrate for Cdh1 and not Cdc20, we speculate that SIRT1 is downregulated at the end of mitotic exit and in the early G1 phase; this hypothesis should be addressed in future studies. Additionally, there is a need to examine whether SIRT1 is subjected to Lys 11-linked polyubiquitination, which is characteristic of APC/C.

Here, we observed the involvement of AROS, an activator of SIRT1, in stress-induced senescence. AROS inhibited SIRT1 ubiquitination by inactivating Cdh1 through competitive interaction with SIRT1.

These findings suggest that AROS could suppress stress-induced cellular senescence by maintaining the abundance of SIRT1. Genome-wide RNA sequencing analysis indicated that AROS regulates the expression patterns of genes associated with the aging process (data not shown). The dependency of AROS-regulated genes on either SIRT1 or Cdh1 should be investigated to verify the functional associations of these factors with gene regulation. For the molecular mechanism underlying AROS function, we propose that AROS and Cdh1 compete for SIRT1, as described above. Additionally, we observed a physical association between AROS and APC2, a subunit of the APC/C complex (data not shown). APC2 functions as a scaffold to form a catalytic subcomplex that contains APC11, APC10/Doc1, and a catalytic coactivator, Cdc20 or Cdh1. Therefore, we speculate that AROS binding to APC2 induces a conformational change in the catalytic core and thereby impedes Cdh1 access to the substrate SIRT1.

Recently, cellular senescence has emerged as an important driver of aging and aging-related diseases, and new senolytic agents for the selective removal of senescent cells are under active investigation. Here, we focused on the potential antisenescence effects of pinosylvin, an analog of resveratrol, as a SIRT1 activator. First, we established bleomycin-induced mouse pulmonary fibrosis as an in vivo senescence system because animal models of stress-induced senescence are scarce. Consistent with the results observed in cellular systems, the expression levels of SIRT1 and AROS were significantly diminished in bleomycin-treated mouse pulmonary tissue, and the addition of pinosylvin provided effective protection against bleomycin-induced pulmonary senescence. The Cdh1 expression patterns showed opposite trends under the same experimental conditions. Cdh1 expression is attenuated by pinosylvin treatment in bleomycin-treated mice but not in PBS-treated mice. Genotoxic stress, such as bleomycin treatment, induces DNA breakage and mediates p53-dependent APC/C-Cdh1 activation^56^, which possibly leads to SIRT1 degradation, whereas pinosylvin may decrease Cdh1 expression to recover the SIRT1 levels. The opposite roles of bleomycin and pinosylvin are reminiscent of those of p53/Cdh1 and SIRT1 in regulating cellular senescence. However, the molecular mechanism underlying the pinosylvin-mediated downregulation of Cdh1 remains to be determined. We also examined the positive correlation between the SIRT1 and AROS protein expression levels using IHC data from a human pulmonary fibrosis tissue microarray (data not shown). Overall, our results suggest opposing roles for Cdh1 and AROS in SIRT1 degradation during the development of pulmonary fibrosis and support pinosylvin as a therapeutic agent for human pulmonary fibrosis. The precise mechanism by which Cdh1 and AROS coordinate the regulation of SIRT1 stability should be addressed in future studies using Cdh1- and AROS-depleted mouse models.

**Fig. 8:**
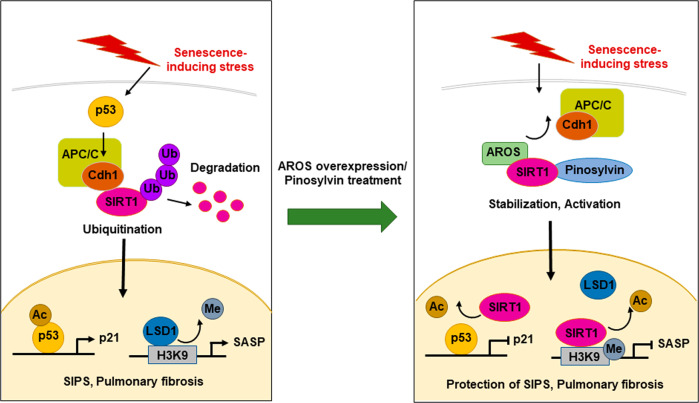
Schematic representation of the roles of AROS and pinosylvin in Cdh1-mediated SIRT1 ubiquitination during stress-induced premature senescence. DNA damage signaling triggers the activation of Cdh1, which leads to SIRT1 degradation and subsequent induction of premature senescence. AROS abrogates SIPS by suppressing Cdh1-mediated SIRT1 ubiquitination and thereby activates SIRT1; these effects cause p53 inactivation and the downregulation of SASP-associated genes. Pinosylvin has a protective effect against bleomycin-induced pulmonary senescence through its function as a SIRT1 activator.

The mechanisms defined in this study are summarized in Fig. 8. In brief, stress exposure induces Cdh1 activation, which leads to SIRT1 ubiquitination and degradation and then the induction of premature senescence. Additionally, AROS, a SIRT1 activator, impairs SIPS by inhibiting Cdh1-mediated SIRT1 ubiquitination. Consequently, the expression levels of genes associated with the p53/p21 pathway and SASP are upregulated. The potential role of LSD1 in the epigenetic activation of the SASP-associated genes *IL-6* and *IL-8* is depicted. As a histone demethylase, LSD1 reduces the levels of the repressive histone marker H3K9me2, leading to upregulation of the expression of SASP genes during DNA damage-induced senescence. After AROS overexpression, SIRT1 is activated, causing the inactivation of p53 and the downregulation of SASP genes through the deacetylation of p53 and histone H3K9ac, respectively. Notably, pinosylvin has a protective effect on bleomycin-induced pulmonary senescence as a SIRT1-activating agent.

## Supplementary information

Supplementary data
