# Supplementary material for: SIRT1 ubiquitination is regulated by opposing activities of APC/C-Cdh1 and AROS during stress-induced premature senescence
Source: Exp Mol Med. 2023 Jun 1;55(6):1232–46. doi: 10.1038/s12276-023-01012-1 (PMC10318011; doi:10.1038/s12276-023-01012-1)
Supplement: Supplementary file 1 — Supplementary data [file 12276_2023_1012_MOESM1_ESM.pdf]

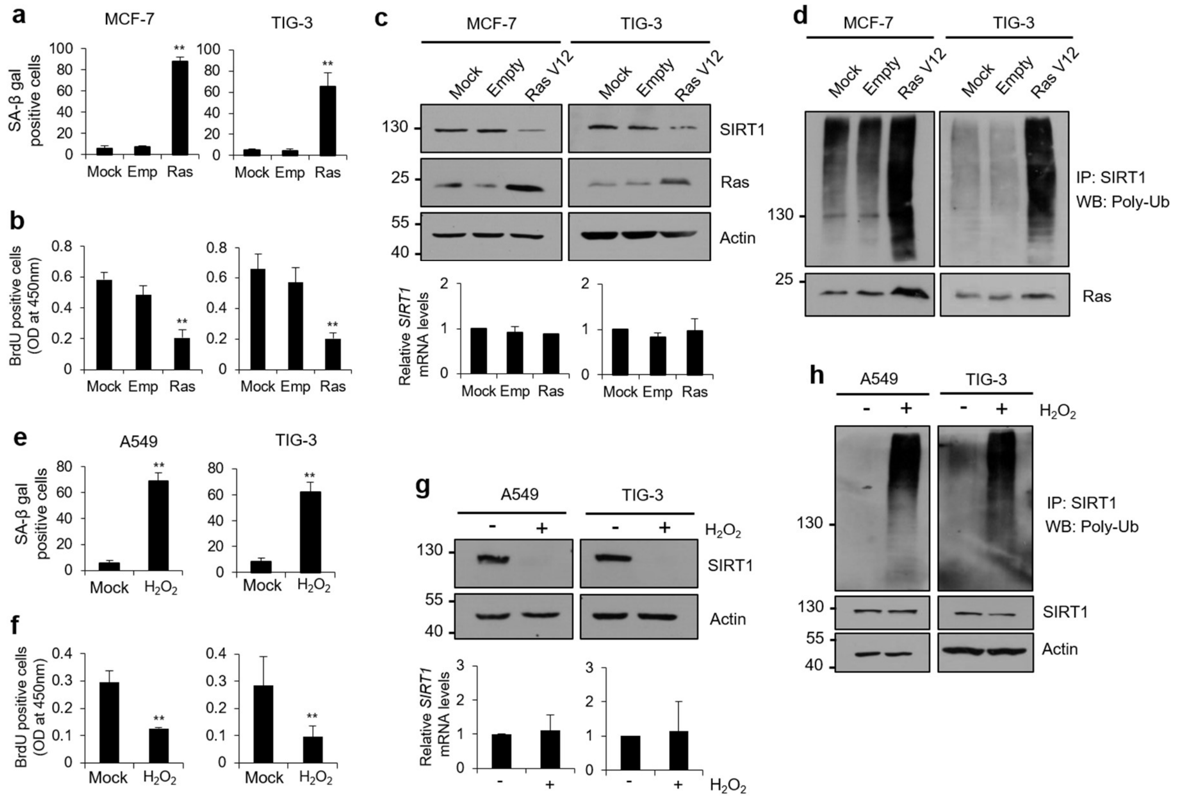

Supplementary Fig. 1 SIRT1 degradation is induced under other stress conditions, Related to Figure 1. a–d Effect of oncogenic *H-RasV12* expression. Both MCF-7 and TIG-3 cells were infected with *H-RasV12* retrovirus for 10 days. Quantification of SA-β-gal-positive cells (**a**). Quantification of BrdU incorporation (**b**). Effect on SIRT1 level. SIRT1 protein and mRNA levels were monitored via WB and RT-qPCR (**c**). Effect on SIRT1 ubiquitination (**d**). The ubiquitination assay was performed as described in Fig. 1G. **e–h** Effect of oxidative stress. Oxidative stress was generated via H_2O_2 treatment of A549 and TIG-3 cells. Cells were treated with 150 μM H_2O_2 for 1 h, then incubated for 5 days after H_2O_2 withdrawal. Quantification of SA-β-gal-positive cells (**e**). Quantification of BrdU incorporation (**f**). Effect on SIRT1 degradation (**g**). Effect on SIRT1 ubiquitination (**h**). Error bars in all panels represent means ± SDs of three independent experiments (**$P < 0.01$).

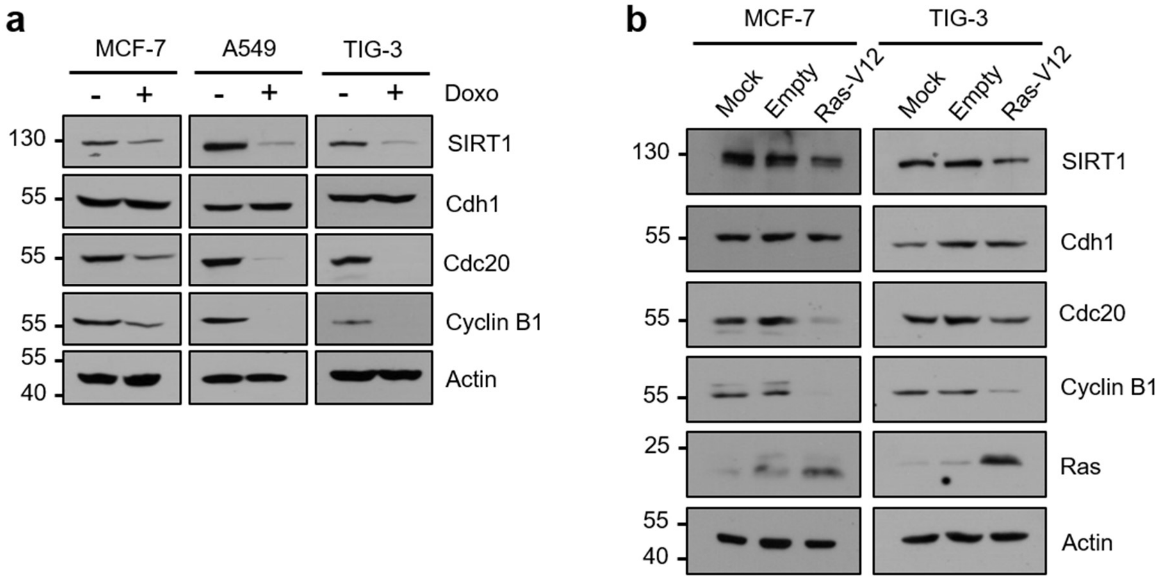

Supplementary Fig. 2-1 Cdh1 substrates, including SIRT1, are downregulated in response to DNA damage and oncogene expression, Related to Figure 2. a Effect of doxorubicin treatment. Cells were treated with doxorubicin, then subjected to WB using antibodies shown on the right. **b** Effect of oncogene expression. MCF-7 and TIG-3 cells were infected with *H-RasV12* retrovirus for 10 days. Cell lysates were subjected to WB analysis using the indicated antibodies.

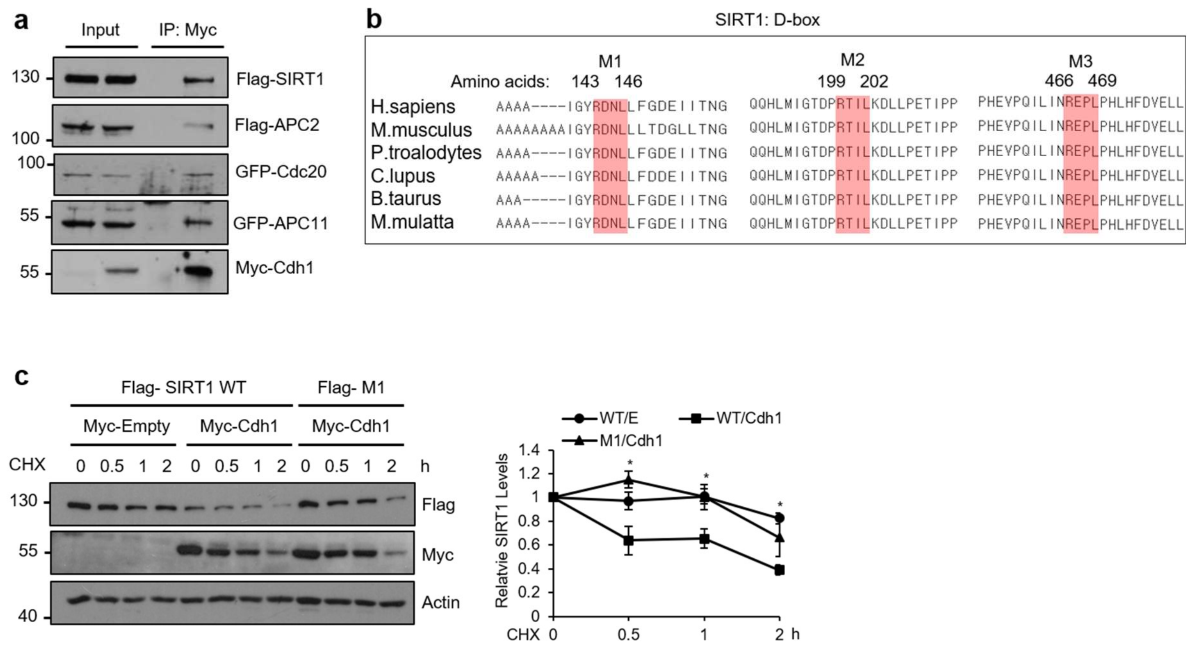

Supplementary Fig. 2-2 First D-box of SIRT1 is required for its ubiquitin-dependent degradation, Related to Figure 2. a Interaction between Cdh1 and SIRT1 under transfection conditions. HEK293 cells were co-transfected with the indicated constructs; lysates were subjected to IP using anti-Myc antibody, followed by WB using anti-Flag or anti-GFP antibody. **b** Alignment of the amino acid sequence of the Destruction box motif (D-box) of SIRT1. Regions corresponding to the putative D-box of SIRT1 in various species are shown. **c** Mutation of the first D-box of SIRT1 impairs Cdh1-mediated SIRT1 turnover. HEK293 cells were co-transfected with the indicated constructs and treated with CHX (50 μg/ml) for the indicated time periods, then subjected to WB using individual antibodies. Error bars represent means ± SDs of three independent experiments (*$P < 0.05$).

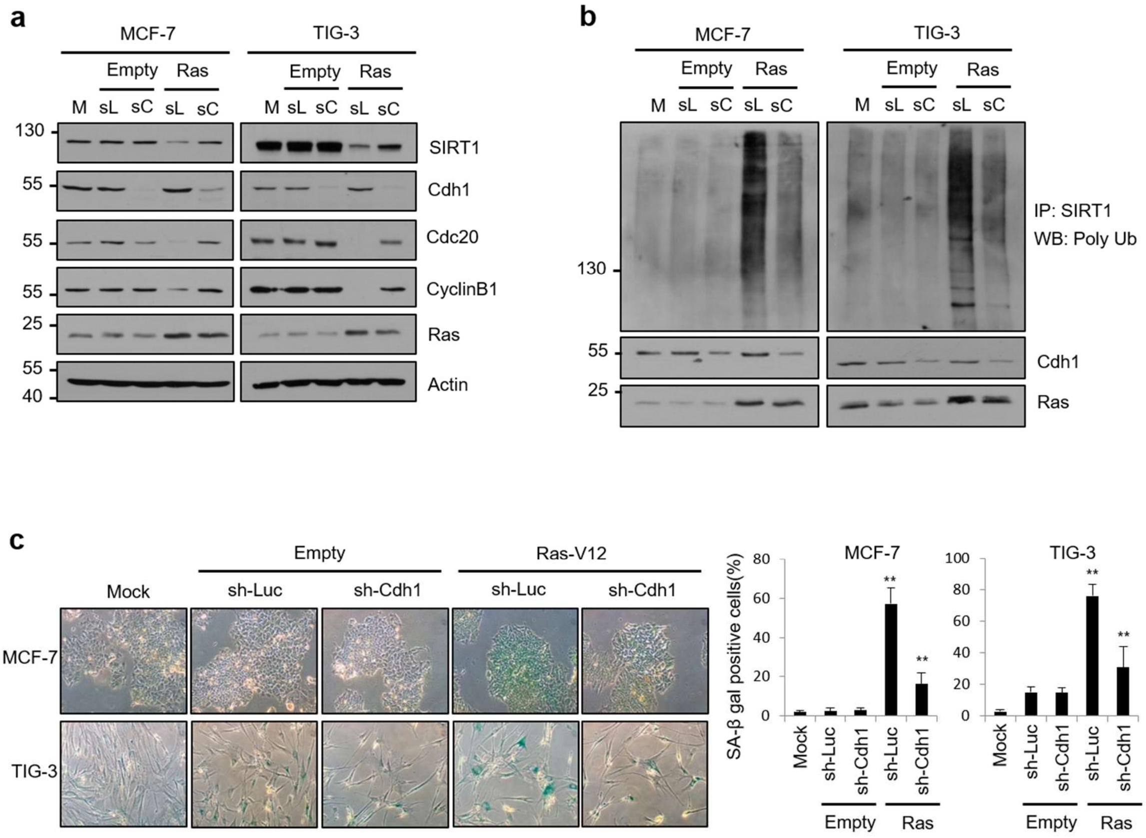

Supplementary Fig. 3 Oncogenic *Ras*-induced senescence and SIRT1 degradation are impaired upon Cdh1 depletion, Related to Figure 3. a–b Both MCF-7 and TIG-3 cells were infected with *H-RasV12* retrovirus before transfection with sh-Cdh1 (sC) or sh-Luc (sL). Cdh1 knockdown and oncogenic *Ras* expression pattern were monitored via WB. Effects of Cdh1 depletion on oncogenic *Ras*-induced SIRT1 degradation (**a**) and SIRT1 ubiquitination (**b**) were assessed via WB using antibodies shown on the right. **c** Effect of Cdh1 knockdown on oncogenic *Ras*-induced senescence. SA-β-gal-positive cells were counted. Error bars represent means ± SDs of three independent experiments (**$P < 0.01$).

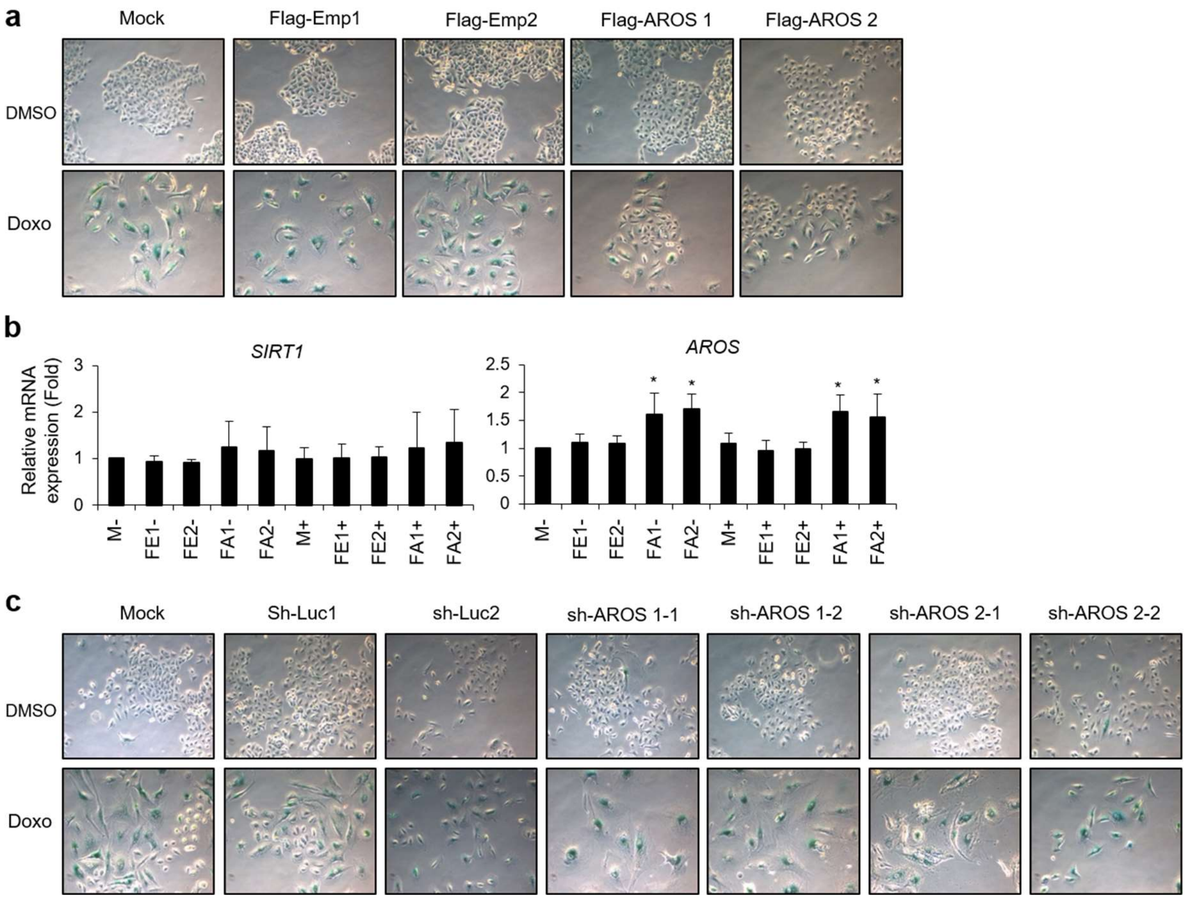

Supplementary Fig. 4 AROS abrogates DNA damage-induced senescence, Related to Figure 4. a Effect of AROS overexpression on doxorubicin-induced senescence. A549 cells, which stably express Flag-AROS, were treated with doxorubicin for 4 days. SA-β-gal-positive cells are shown. **b** Effect of AROS overexpression on SIRT1 mRNA expression level, analyzed via RT-qPCR. Error bars represent means ± SDs of three independent experiments (*$P < 0.05$). **c** Effect of AROS knockdown on doxorubicin-mediated senescence. SA-β-gal-positive cells were visualized via microscopy. Abbreviations: M, Mock; FE1, Flag-Empty #1; FE2, Flag-Empty #2; FA1, Flag-AROS #1; FA2, Flag-AROS #2.

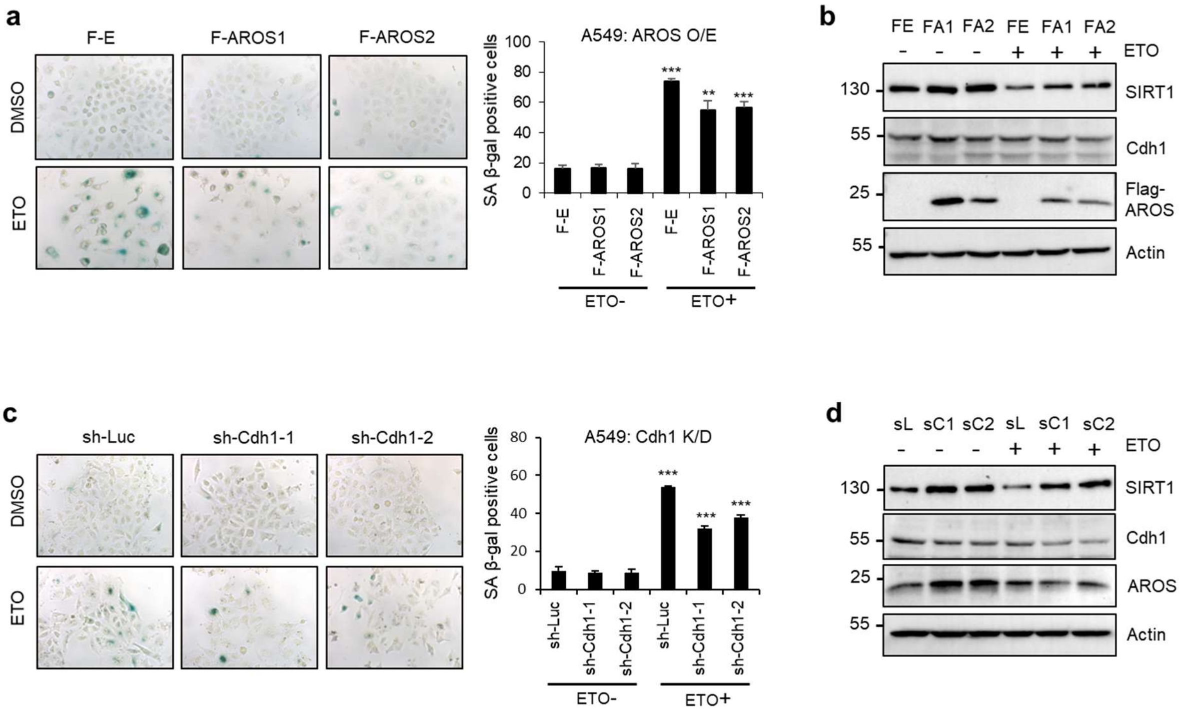

Supplementary Fig. 5-1 AROS and Cdh1 regulate SIRT1 turnover during etoposide-induced senescence, Related to Figures 3 and 4. a–b Effects of AROS overexpression on etoposide-induced senescence (**a**), and SIRT1 degradation (**b**). 1 μM etoposide was applied to two subclones of A549 cells (F-AROS1 and F-AROS2) that stably express Flag-AROS. **a** After 48 h, cells were examined via SA-β-gal staining. Error bars represent means ± SDs of three independent experiments (**$P < 0.01$, ***$P < 0.001$). **b** Under the same conditions, SIRT1 levels were monitored *via* WB using the indicated antibodies. **c–d** Effects of Cdh1 knockdown on etoposide-induced senescence (**c**), and SIRT1 degradation (**d**). **c** After 48 h, cells were examined via SA-β-gal staining. Error bars represent means ± SDs of three independent experiments (***$P < 0.001$). **d** Under the same conditions, SIRT1 levels were monitored by WB using the indicated antibodies.

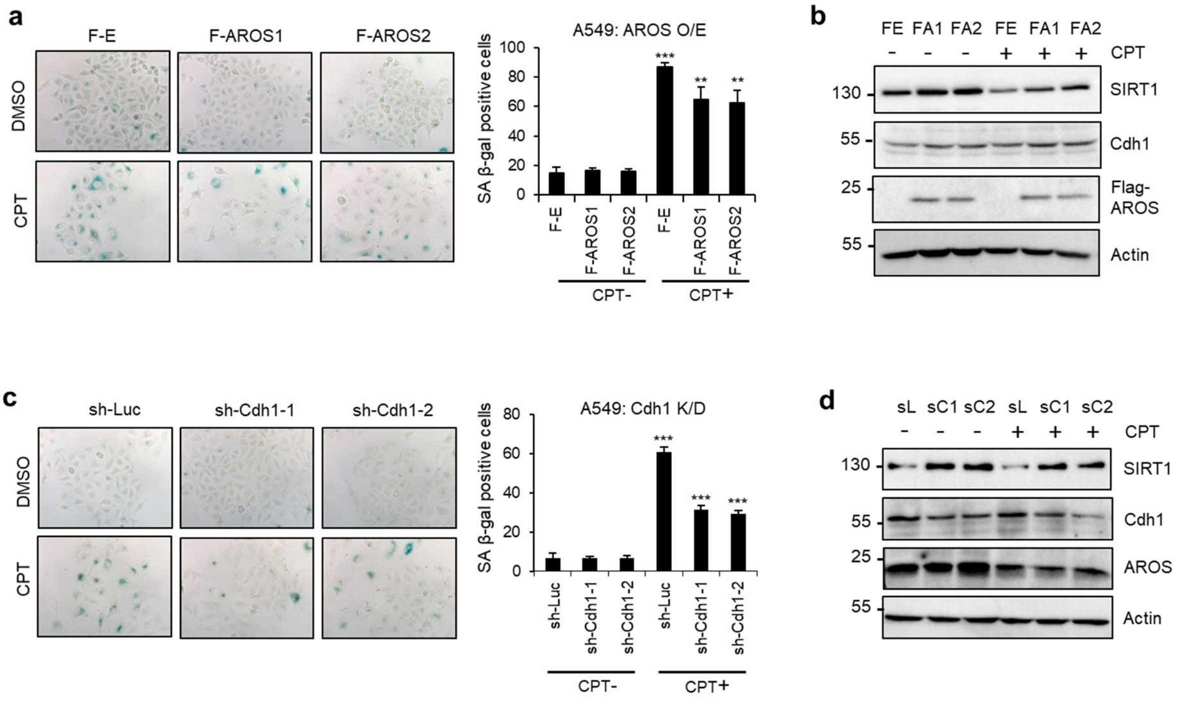

Supplementary Fig. 5-2 AROS and Cdh1 regulate SIRT1 turnover during camptothecin-induced senescence, Related to Figures 3 and 4. a–b Effects of AROS overexpression on camptothecin-induced senescence (**a**), and SIRT1 degradation (**b**). 10 nM camptothecin was applied to two subclones of A549 cells (F-AROS1 and F-AROS2) that stably express Flag-AROS. **a** After 4 days, cells were examined via SA-β-gal staining. Error bars represent means ± SDs of three independent experiments (***P* < 0.01, ****P* < 0.001). **b** Under the same conditions, SIRT1 levels were monitored via WB using the indicated antibodies. **c–d** Effects of Cdh1 knockdown on camptothecin-induced senescence (**c**), and SIRT1 degradation (**d**). **c** After 4 days, cells were examined via SA-β-gal staining. Error bars represent means ± SDs of three independent experiments (****P* < 0.001). **d** Under the same conditions, SIRT1 levels were monitored via WB using the indicated antibodies.

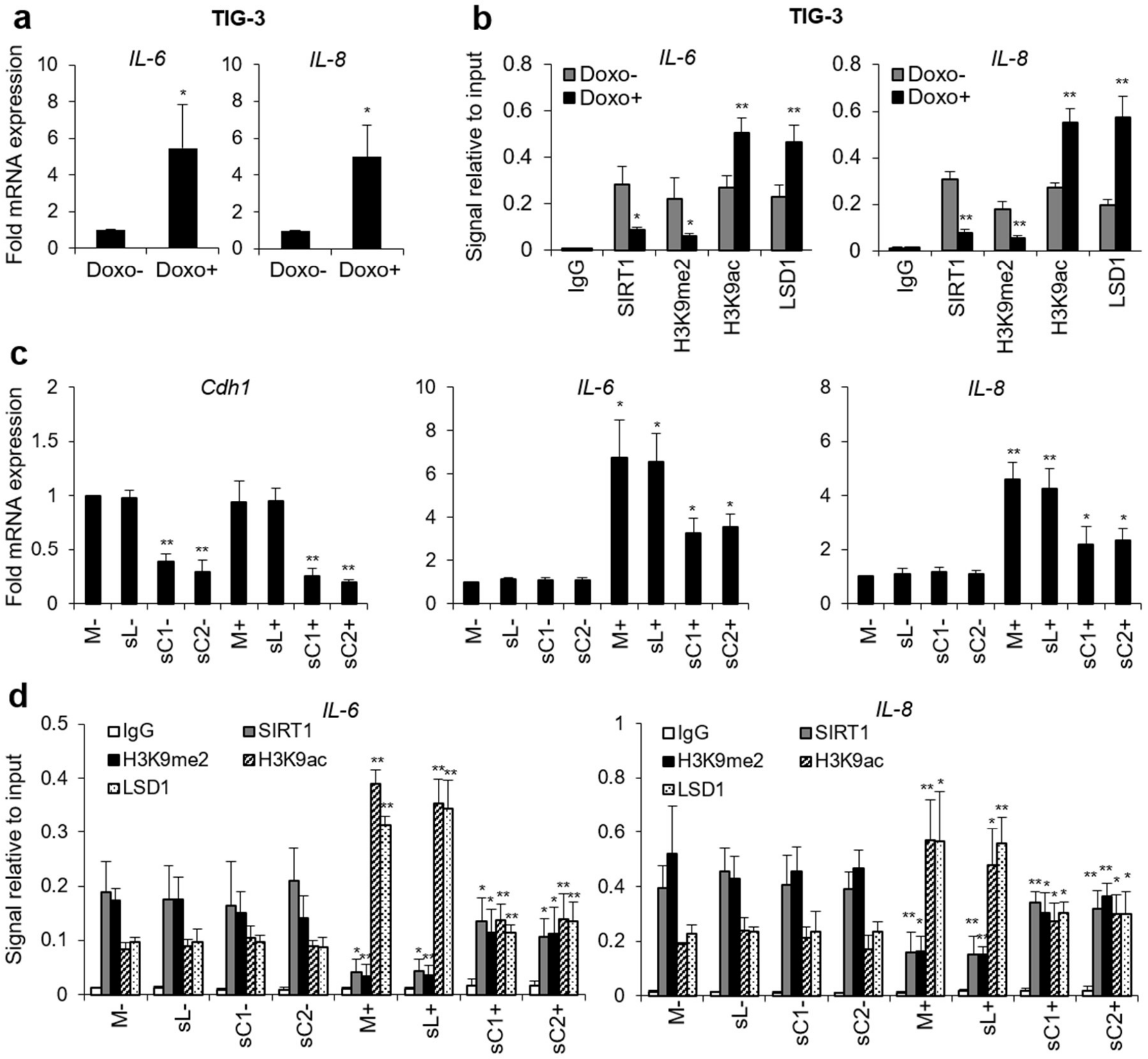

a
TIG-3
IL-6
IL-8
Fold mRNA expression
Doxo-
Doxo+
b
TIG-3
IL-6
IL-8
Signal relative to input
Doxo-
Doxo+
IgG
SIRT1
H3K9me2
H3K9ac
LSD1
c
Cdh1
IL-6
IL-8
Fold mRNA expression
M-
sL-
sC1-
sC2-
M+
sL+
sC1+
sC2+
d
IL-6
IL-8
Signal relative to input
IgG
SIRT1
H3K9me2
H3K9ac
LSD1

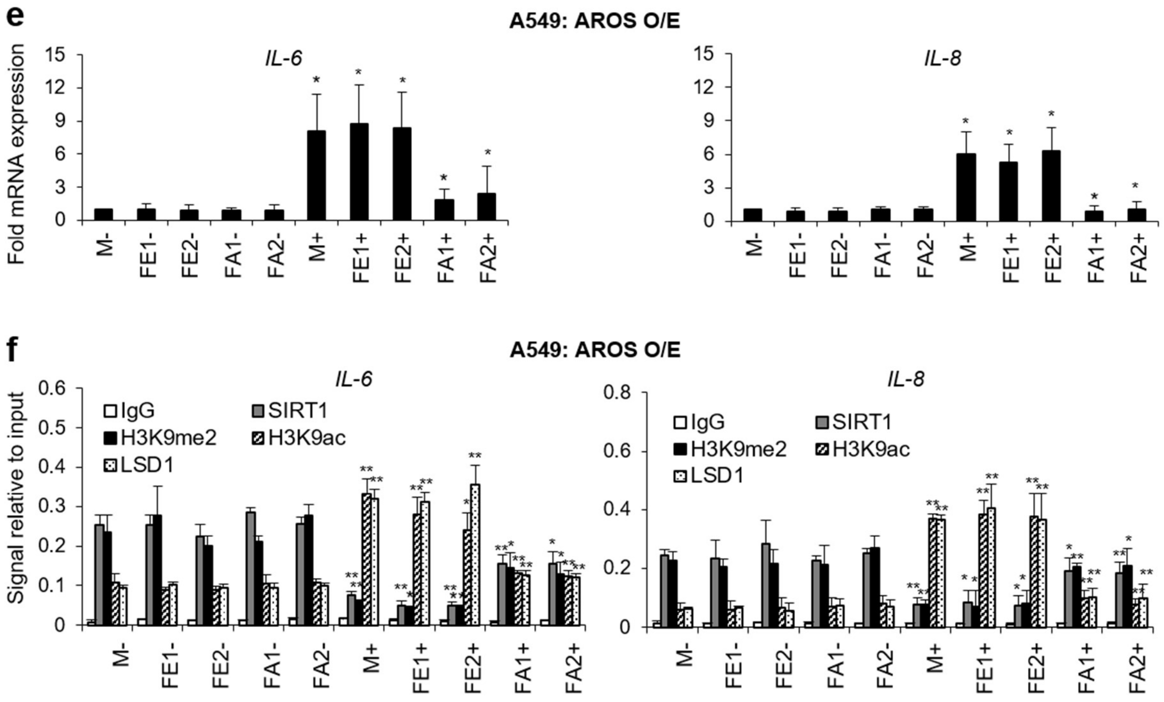

Supplementary Fig. 6 Effect of Cdh1 knockdown and AROS overexpression on the epigenetic regulation of SASP-associated genes, Related to Figure 6. a–d All experiments were conducted as described in Figure 6a–6d, using TIG-3 cells instead of A549 cells. **e** Effect of AROS overexpression on *IL-6* and *IL-8* mRNA expression patterns. Two clones of A549 cells with stable AROS overexpression (FA1 and FA2) were treated with (+) and without (–) doxorubicin, then subjected to RT-qPCR analysis. **f** Effect of AROS overexpression on epigenetic regulation of SASP genes. Stable cells were treated with (+) doxorubicin, then subjected to qChIP analysis using the indicated antibodies. Abbreviations: M, Mock; FE1, Flag-Empty #1; FE2, Flag-Empty #2; FA1, Flag-AROS #1; FA2, Flag-AROS #2. Error bars in all panels represent means ± SDs of three independent experiments (*$P < 0.05$, **$P < 0.01$)

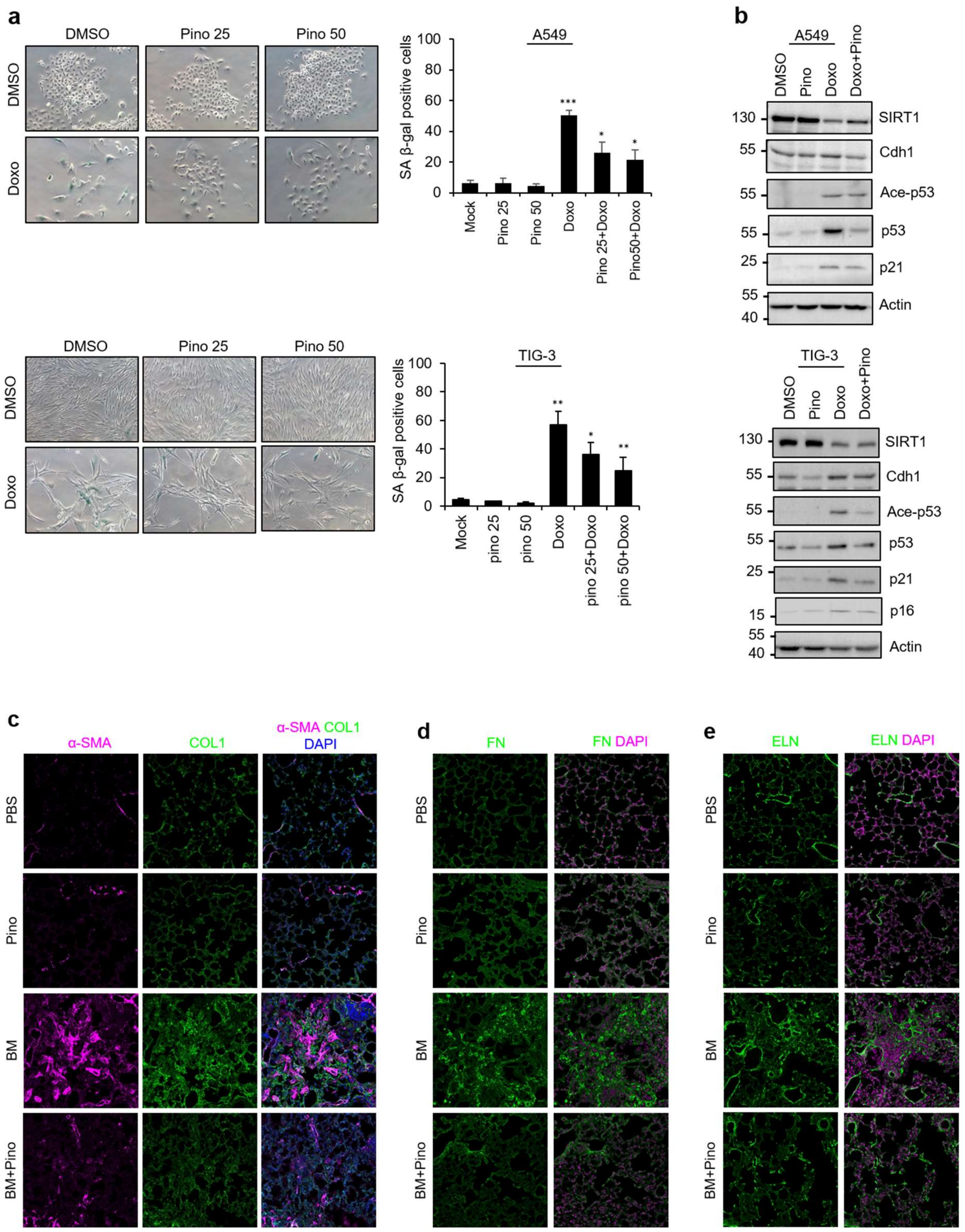
a
DMSO
Pino 25
Pino 50
DMSO
Doxo
A549
SA β-gal positive cells
Mock
Pino 25
Pino 50
Doxo
Pino 25+Doxo
Pino50+Doxo
TIG-3
pino 25
pino 50
pino 25+Doxo
pino 50+Doxo
b
A549
DMSO
Pino
Doxo
Doxo+Pino
SIRT1
Cdh1
Ace-p53
p53
p21
Actin
TIG-3
p16
c
α-SMA
COL1
α-SMA COL1 DAPI
PBS
Pino
BM
BM+Pino
d
FN
FN DAPI
e
ELN
ELN DAPI

Supplementary Fig. 7 Pinosylvin reverses doxorubicin-induced senescence by upregulating SIRT1, Related to Figure 7. a Pinosylvin inhibits doxorubicin-induced senescence in A549 and TIG-3 cells. Senescent cells were monitored and quantified via SA-β-gal staining. **b** Pinosylvin suppresses the p53-p21 and p16 senescence pathways in doxorubicin-mediated senescent cells. Protein expression patterns were detected via WB using the indicated antibodies. All data represent means ± SDs of three independent experiments. Significant changes are indicated (*$P < 0.05$, **$P < 0.01$, ***$P < 0.001$). **c**–**e** Immunostaining for fibrosis markers α-SMA, Collagen Type I (Col I) (**c**), Fibronectin (FN) (**d**), and Elastin (ELN) (**e**) in PBS, Pino, BM, and BM/Pino-administered lung sections.
